# A synergistic acid–base tandem co-sensitization approach using pyrimidine fluorescent dyes achieves 22% indoor efficiency

**DOI:** 10.1038/s41598-026-40785-z

**Published:** 2026-03-23

**Authors:** Safa A. Badawy, Wael Shehta, Asmaa A. Masry, Ahmed H. Moustafa, Mohamed R. Elmorsy

**Affiliations:** 1 Department of Chemistry, Faculty of Science, Mansoura University, Mansoura, 35516 Egypt; 2 Chemistry Department, Faculty of Science, Zagazig University, Zagazig, 44519 Egypt

**Keywords:** Dye-sensitized solar cells, Fluorescent dyes, Acid–base co-sensitization, Pyrimidine, N3 dye, Tandem DSSC, Photostability, Chemistry, Energy science and technology, Engineering, Materials science, Optics and photonics

## Abstract

In this research, an integrated acid–base tandem co-sensitization approach was formulated to improve the photovoltaic efficiency of dye-sensitized solar cells (DSSCs) under both indoor and outdoor lighting conditions. Novel pyrimidine- fluorescent based organic co-sensitizers **(AS-1–AS-4)** were systematically combined with the Ru(II)-based **N3** dye to investigate their synergistic effect on light harvesting, charge transfer, and long-term stability. The optimized tandem configuration **(AS-1 (bottom) + N3 (top))** demonstrated a remarkable 68% improvement in overall efficiency compared to the single **N3** reference cell, achieving 11.12% under AM 1.5G illumination and 22.02% under 1000 lx indoor light. This significant enhancement is attributed to complementary spectral absorption, efficient acid–base interfacial coupling, and suppressed charge recombination, as confirmed by UV–Vis, EIS, IPCE, and stability analyses. The findings demonstrate that the acid–base tandem co-sensitization technique offers a highly effective and promising route for developing next-generation dye-sensitized solar cells (DSSCs) with superior efficiency, stability, and performance under low-light conditions.

**Supplementary Information:**

The online version contains supplementary material available at 10.1038/s41598-026-40785-z.

## Introduction

**Fig. 1:**
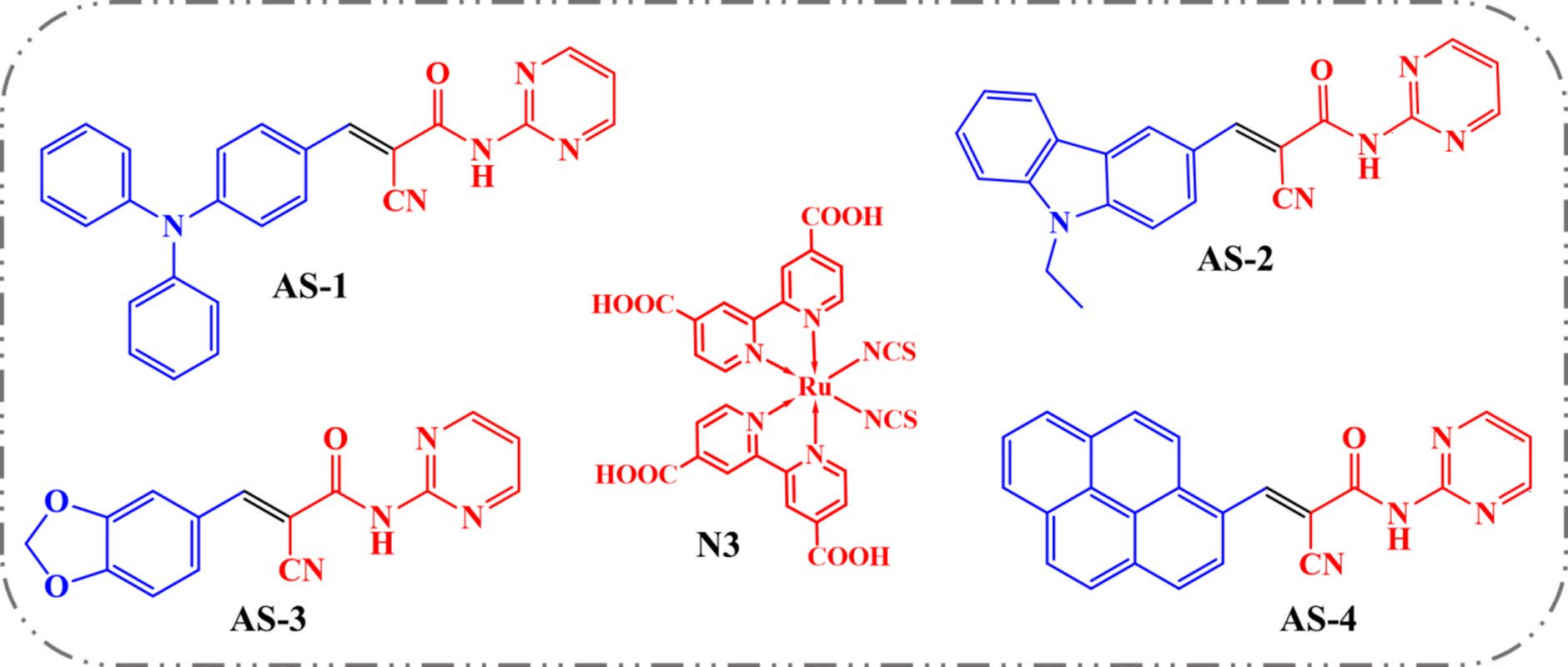
Molecular structures of co-sensitizers **AS-1-4** and **N3.**

The rapid expansion of the global population has accelerated the depletion of non-renewable fossil energy resources, thereby amplifying the need for sustainable and renewable energy technologies such as photovoltaic, hydro, wind, thermal, and biomass systems to secure future energy supplies^1^. These renewable approaches not only minimize environmental degradation but also provide viable routes for scalable and cost-efficient power generation^2^. Among the diverse photovoltaic technologies, dye-sensitized solar cells (DSSCs), first introduced by Grätzel and colleagues in 1991, have attracted significant attention due to their straightforward fabrication process, eco-friendly nature, and low production cost, positioning them as strong contenders to complement traditional silicon-based solar cells^3^. A typical DSSC comprises four essential components: (i) a photoanode of a wide-band-gap semiconductor (usually TiO_2_), (ii) a light-harvesting sensitizer, (iii) a redox electrolyte for dye regeneration, and (iv) a counter-electrode^4^. Upon illumination, the sensitizer absorbs photons and injects electrons into the conduction band of TiO_2_; the oxidized dye is subsequently regenerated by the redox couple in the electrolyte^5^. Hence, the sensitizer plays a pivotal role in governing the device’s efficiency through its light-harvesting, charge-transfer, and regeneration capabilities. Ruthenium(II) polypyridyl complexes, including **N3**, **N719**, **Z907**, and **NCSU-10**, have long been benchmark dyes in DSSCs because of their outstanding photophysical stability, strong metal-to-ligand charge-transfer (MLCT) absorption, and optimal redox potentials^6^. The N3 dye in particular exhibits excellent chemical stability, broad visible absorption, and efficient electron injection into TiO_2_, leading to power-conversion efficiencies above 10% and long-term operational durability^7^. Moreover, **N3** has been successfully employed as an acidic component in acid–base co-sensitization systems, where its carboxylate groups preferentially anchor to Brønsted-acid sites on TiO_2_^8^. Despite these advantages, the scarcity, cost, and multistep synthesis of Ru-based dyes have stimulated research into metal-free organic sensitizers such as phenothiazine, carbazole, triphenylamine, and benzofuran derivatives, which offer facile structural tunability, low environmental impact, and high molar absorptivity^9–12^. These organic donor–π–acceptor (D–π–A) molecules can be engineered to optimize the alignment of frontier molecular orbitals, enabling efficient intramolecular charge transfer (ICT) and extended spectral absorption. While such dyes may suffer from aggregation or limited regeneration in certain electrolytes, their absorption versatility allows effective spectral complementarity when combined with Ru dyes^13^. Co-sensitization, which involves employing two or more dyes with mutually complementary absorption profiles, is a well-recognized approach to attaining panchromatic light absorption and improved TiO_2_ surface coverage^14,15^. This method improves both incident-photon-to-current efficiency (*IPCE*) and short-circuit current density (*Jsc*). The approach becomes particularly powerful in acid–base systems, where different dyes selectively adsorb onto distinct surface sites of TiO_2_, Brønsted and Lewis acid centers thereby suppressing competitive adsorption and minimizing electron recombination^16,17^. Anchoring groups such as rhodanine-3-acetic acid and carboxylate moieties strengthen interfacial coupling, while nitrogen-containing heterocycles (pyridyl, pyrimidine, or pyrimidine) coordinate to Lewis-acidic Ti⁴⁺ centers, establishing complementary adsorption and efficient charge transport^18–20^. Recent studies have demonstrated that nitrogen-rich heteroaromatic acceptors can extend π-conjugation, facilitate selective TiO_2_ binding, and promote enhanced light harvesting across the visible spectrum^21,22^. Building on this concept, the present work introduces a new family of fluorescent pyrimidine-based co-sensitizers **AS-1–AS-4** as shown in Fig. 1, featuring varied donor units, triphenylamine, ethyl-carbazole, piperonal, and pyrene coupled to a common pyrimidine acceptor. When integrated with the Ru-based **N3** dye in an acid–base tandem configuration, these dyes deliver complementary spectral absorption and improved interfacial charge transfer. The optimized AS-1 (bottom)/N3 (top) tandem configuration achieves up to a 68% enhancement in overall power-conversion efficiency relative to the single **N3** system, with efficiencies reaching 11.12% (AM 1.5 G) and 22.02% under 1000 lx indoor illumination. This significant improvement arises from the synergistic effects of acid–base dual-site adsorption, broadened light harvesting, and suppressed electron recombination. The results establish acid–base tandem fluorescent co-sensitization as an effective route toward high-performance, durable DSSCs operable under both indoor and outdoor conditions.

## Experimental

Detailed photovoltaic performance data for the co-sensitized devices based on **AS-1–4 + N3** dyes, as well as for the tandem device fabricated using **AS-1 (bottom layer) and N3 (top layer)**, are presented and discussed in the supplementary file. Also, the UV–Vis absorption spectrum of **N3** is provided in Figure S1. Comprehensive structural and spectroscopic characterization of the **AS-1–4** sensitizers, including all relevant analyses, is compiled in Figures S2–S22 and is available in the Supporting Information.

### Synthesis of 2-cyano-N-(pyrimidin-2-yl)acetamide (1)

A mixture of pyrimidin-2-amine and 1-(cyanoacetyl)-3,5-dimethylpyrazole were refluxed for 4 h in dioxane, the melting point for the solid product which formed were as literature reported^23^.

### General synthesis of organic sensitizers **AS1-4**

In a dry 50 mL round-bottom flask containing compound (**1)**, 4-(diphenylamino)benzaldehyde (0.54 g, 2 mmol) **(2)**, 9-ethyl-9*H*-carbazole-3-carbaldehyde (0.44 g, 2 mmol) **(3)**, benzo[*d*][1,3] dioxole-5-carbaldehyde (0.30 g, 2 mmol) **(4)** and pyrene-1-carbaldehyde (0.46 g, 2 mmol) **(5)** were dissolved in 20 mL ethanol with 1–2 mL of acetic acid were added. The reaction mixture was heated at 80 ˚C for 3 h reflux and the reaction was monitored by thin-layer chromatography (TLC). The solid product in each case was filtered and washed and recrystallized by ethanol to obtain sensitizers **AS-1-4**.

### 2-Cyano-3-(4-(diphenylamino)phenyl)-N-(pyrimidin-2-yl)acrylamide (**AS-1**)

Orange crystal (85% yield); m.p. = 280–282 °C. IR (KBr) *ν*_*max*_.cm^− 1^: 3393 (N-H), 2922 (C-H aliphatic), 2196 (C ≡ N), 1668 (C = O). ^1^H NMR (DMSO-*d*_*6*_, ppm): δ 6.94 (t, *J* = 8.00 Hz, 2 H, Ar-H), 7.09 (d, *J* = 8.00 Hz, 4 H, Ar-H), 7.20–7.31 (m, 7 H, Ar-H), 7.36–7.39 (t, *J* = 8.00 Hz, 2 H, Ar-H), 7.54 (s, 1H, =CH), 8.73 (d, *J* = 4.00 Hz, 2 H, pyrimidinyl-H). ^13^C NMR δ: 106.8, 116.5, 117.5, 124.9 (3 C), 125.8, 126.2, 126.3 (4 C), 129.2, 129.9 (4 C), 136.2, 145.5 (2 C), 146.6, 151.2, 155.3 (2 C), 156.0 and 170.7 ppm. Mass analysis (m/z, %): 417 (M^+^, 27.82), 401 (46.82), 317 (38.19), 263 (100.0), 203 (76.30), 157 (73.53), 120 (52.15), 89 (63.62). Analysis calcd. For C_26_H_19_N_5_O (417.16): C, 74.80; H, 4.59; N, 16.78% Found: C, 73.18; H, 4.46; N, 17.01%.

### 2-Cyano-3-(9-ethyl-9 H-carbazol-3-yl)-N-(pyrimidin-2-yl)acrylamide (**AS-2**)

Yellowish crystal (83% yield); m.p. = 220–222 °C. IR (KBr) *ν*_*max*_.cm^− 1^: 3445 (N-H), 3129 (C-H aromatic), 2997 (C-H aliphatic), 2215 (C ≡ N), 1652 (C = O). ^1^H NMR (DMSO-*d*_*6*_, ppm): δ 1.35 (t, *J* = 7.50 Hz, 3 H, CH_3_), 4.33 (q, *J* = 7.50 Hz, 2 H, CH_2_), 7.21–7.33 (m, 4 H, Ar-H), 7.38 (d, *J* = 8.00 Hz, 1H, Ar-H), 7.43 (d, *J* = 8.00 Hz, 1H, Ar-H), 7.59 (s, 1H, Ar-H), 7.63 (d, *J* = 8.00 Hz, 1H, Ar-H), 8.02 (s, 1H, =CH), 8.55 (s, 1H, N-H), 8.74 (d, *J* = 4.00 Hz, 2 H, pyrimidinyl-H), ^13^C NMR δ: 13.3, 42.6, 105.0, 106.7, 109.0, 116.4, 117.4, 122.0, 123.9, 126.0, 128.4, 129.1 (2 C), 139.7, 141.7, 151.1 (2 C), 155.2 (3 C), 155.9 and 170.6 ppm. Mass analysis (m/z, %): 366 (M^+^, 22.82), 310 (14.25). 264 (50.20), 100 (27.53), 86 (100.00), 82 (59.28). Analysis calcd. For C_22_H_17_N_5_O (366.41): C, 71.92; H, 4.66; N, 19.06%. Found: C, 71.29; H, 4.96; N, 18.90%.

### 3-(Benzo[d][1,3]dioxol-5-yl)-2-cyano-N-(pyrimidin-2-yl)acrylamide (**AS-3**)

Yellow crystal (93% yield); m.p. = 238–240 °C. IR (KBr) *ν*_*max*_.cm^− 1^: 3435 (N-H), 3169 (C-H aromatic), 2971, (C-H aliphatic), 2220 (C ≡ N), 1680 (C = O). ^1^H NMR (DMSO-*d*_*6*_, ppm): δ 6.70 (s, 2 H, CH_2_ ), 7.74 (d, *J* = 8.00 Hz, 1H, Ar-H), 7.90 (t, *J* = 8.00 Hz, 2 H, Ar-H), 8.09 (t, *J* = 8.00 Hz, 1H, Ar-H), 8.35 (s, 1H, =CH), 9.28 (s, 1H, N-H), 9.54 (d, *J* = 4.00 Hz, 2 H, pyrimidinyl-H). ^13^C NMR: δ 102.2, 106.9, 109.8, 110.9, 116.6, 117.5, 124.1, 128.2, 148.9, 150.2, 151.3, 155.4 (2 C), 156.0 and 170.8 ppm. Mass analysis (m/z, %): 294 (M^+^, 11.99), 223 (29.66), 162 (43.29), 109 (68.75), 85 (89.85), 68 (98.26), 57 (100.0). Analysis calcd. For C_15_H_10_N_4_O_3_ (294.27): C, 61.22; H, 3.43; N, 19.04% Found: C, 61.18; H, 4.10; N, 18.82%.

### 2-Cyano-3-(pyren-1-yl)-N-(pyrimidin-2-yl)acrylamide (**AS-4**)

Yellow crystal (94% yield); m.p. = 262–264 °C. IR (KBr) *ν*_*max*_.cm^− 1^: 3449 (N-H), 2973 (C-H aliphatic), 2199 (C ≡ N), 1689 (C = O). ^1^H NMR (DMSO*-d*_*6*_, *ppm*): δ 7.26 (t, *J* = 8.00 Hz, 1H, Ar-H), 7.63 (t, *J* = 8.00 Hz, 1H, Ar-H), 7.69 (d, *J* = 8.00 Hz, 3 H, Ar-H), 7.75 (d, *J* = 8.00 Hz, 1H, Ar-H), 7.79 (d, *J* = 8.00 Hz, 2 H, Ar-H), 7.94 (d, *J* = 4.00 Hz, 1H, pyrimidinyl-H), 8.04 (d, *J* = 4.00 Hz, 1H, pyrimidinyl-H), 8.48 (s, 1H, =CH), 8.69 (d, *J* = 8.00 Hz, 2 H, Ar-H), 9.57 (s, 1H, N-H), ^13^C NMR: δ 108.3, 116.4, 117.4, 123.7, 124.7, 125.4, 125.7 (2 C), 126.4, 127.2, 127.6, 128.2, 128.3, 128.9 (2 C), 129.0 (2 C), 130.2, 130.9, 131.7, 148.7, 155.2, 155.9 and 170.6 ppm. Mass analysis (m/z, %): 374 (M^+^, 11.25), 354.74 (30.93), 229 (52.22), 129 (46.52), 96 (100.00), 95 (73.67), 83 (37.32). Analysis calcd. For C_24_H_14_N_4_O (374.40): C, 76.99; H, 3.77; N, 14.96% Found: C, 76.18; H, 4.01; N, 14.12%.

## Results and discussions

### Synthesis

**Scheme 1:**
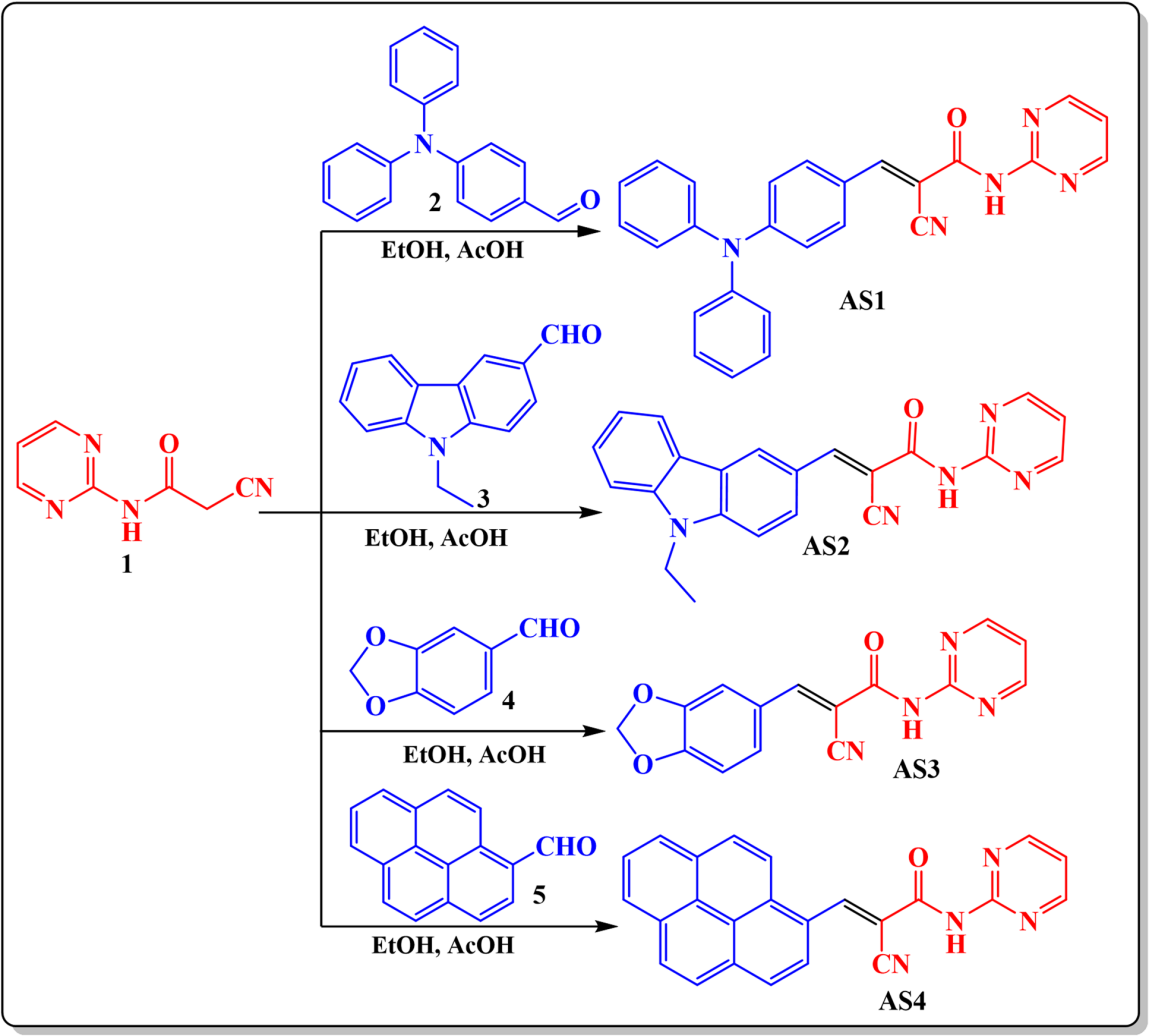
Synthesis pathway of pyrimidine sensitizers **AS1-4.**

2-Cyano-*N*-(pyrimidin-2-yl)acetamide **(1)** was synthesized following a previously reported procedure^23^, in which pyrimidin-2-amine was refluxed with 1-(cyanoacetyl)-3,5-dimethylpyrazole in dioxane to yield the desired compound^23^. As shown in Scheme 1, the synthesis involves acid-catalyzed Knoevenagel condensation of 2-cyano-*N*-(pyrimidin-2-yl)acetamide (**1**) with different aromatic aldehyde derivatives (namely; 4-(diphenylamino)benzaldehyde **(2)**, 9-ethyl-9*H*-carbazole-3-carbaldehyde **(3)**, benzo[*d*][1,3] dioxole-5-carbaldehyde **(4)**, and pyrene-1-carbaldehyde **(5).** The condensation reaction was carried out in a boiling solution of EtOH and in the presence of acetic acid as a catalyst to afford desired sensitizers **AS1-4** (Scheme 1). **AS-1** was obtained as orange crystals (85% yield, m.p. = 280–282 °C), consistent with the extended conjugation of its triphenylamine donor. The IR spectrum exhibited prominent bands at 3393 cm^− 1^ (N-H stretching), 2196 cm⁻¹ (C ≡ N stretching), and 1668 cm^− 1^ (C = O stretching), confirming the amide and cyano functionalities. The ^1^H NMR spectrum showed significant singlet signal assigned to the vinylic = CH proton at δ 7.54 ppm and doublet signals at δ 8.73 ppm (2 H) was attributed to the pyrimidinyl protons, supporting the successful conjugation between donor and acceptor units. The ^13^C NMR data revealed signals at **δ** 116–170 ppm, including the characteristic cyano carbon near δ 116.5 ppm and carbonyl (C = O) at δ 170.7 ppm. **AS-2**, containing an ethylcarbazole donor, crystallized as yellowish crystals (83% yield, m.p. = 220–222 °C). The IR spectrum displayed absorption bands at 3445 cm⁻¹ (N-H), 2215 cm⁻¹ (C ≡ N), and 1652 cm^− 1^ (C = O). The ^1^H NMR spectrum featured characteristic ethyl group signals at δ 1.35 ppm (t, 3 H) and δ 4.33 ppm (q, 2 H), confirming N-ethyl substitution. Aromatic protons resonated between δ 7.21–7.63 ppm, while the vinylic = CH appeared at δ 8.02 ppm, and the amide N–H at δ 8.55 ppm. The downfield doublet at δ 8.74 ppm (2 H) was attributed to the pyrimidine moiety. The ^13^C NMR spectrum exhibited signals at δ 13.33 and 42.69 ppm for the ethyl substituent, and cyano and carbonyl carbons at δ 116.4 and δ 170.6 ppm, respectively. The analytical results closely matched the theoretical composition, confirming C₂₂H₁₇N₅O purity. **AS-3**, synthesized using piperonal aldehyde, was obtained as yellow crystals (93% yield, m.p. = 238–240 °C**)**. The IR spectrum displayed absorptions at 3435 cm⁻^−1^ (N–H), 2220 cm⁻¹ (C ≡ N), and 1680 cm^− 1^ (C = O). The ^1^H NMR spectrum showed a distinctive singlet at δ 6.70 ppm, attributed to the methylenedioxy protons (–O–CH₂–O–) of the piperonal ring. The amide proton appeared as a sharp singlet at δ 9.28 ppm, and the vinylic = CH signal was observed at δ 8.35 ppm, and the protons of pyrimidine moiety appeared as doublet signal at 9.54 ppm appeared. **AS-4**, featuring a pyrene donor, afforded yellow crystals (94% yield, m.p. = 262–264 °C). The IR spectrum exhibited characteristic bands at 3449 cm^− 1^ (N-H), 2199 cm⁻¹ (C ≡ N), and 1689 cm^− 1^ (C = O). The ^1^H NMR spectrum revealed multiple aromatic signals between δ 7.26–8.69 ppm corresponding to the pyrene skeleton, with a singlet at δ 8.48 ppm assigned to the vinylic = CH proton. The amide proton appeared at δ 9.57 ppm, consistent with hydrogen-bonded N-H.

### Photophysical properties

**Fig. 2:**
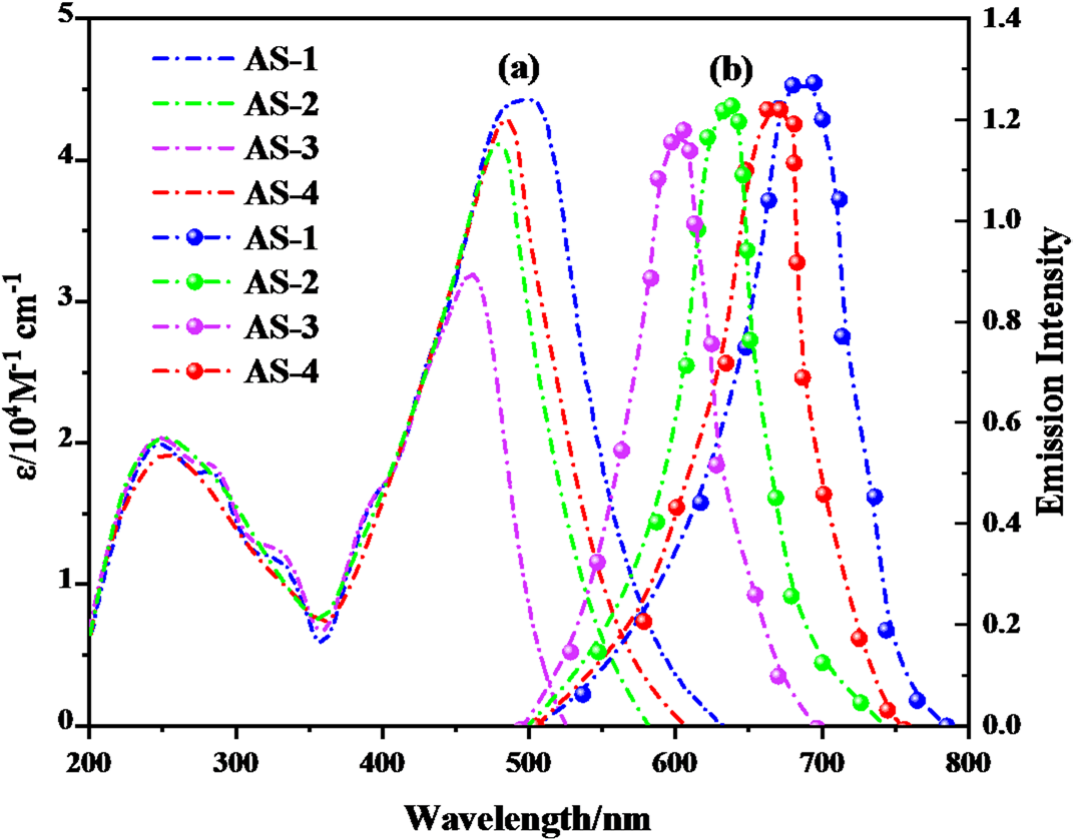
(**a**) UV-Vis absorption and (**b**) emission spectra of **AS-1-4** sensitizers.

The optical absorption and emission characteristics of the pyrimidine-based co-sensitizers **AS-1**–**AS-4** were examined in DMF (2 × 10^− 5^ M), and the corresponding spectra are presented in Fig. 2a–b, with key parameters summarized in Table 1.

**Table 1:** The optical absorption and emission characteristics of the pyrimidine-based co-sensitizers **AS-1**–**AS-4.**

Sensitizer	λ_max_/ nm	ε/10^4^M^− 1^ cm^− 1^	λ_em_^b^ /nm	Experimental E_0−0_ (eV)
**AS-1**	504	4.41	691	2.12
**AS-2**	480	4.14	638	2.26
**AS-3**	463	3.16	601	2.39
**AS-4**	483	4.29	665	2.19

^a^Absorption and ^b^Emission spectra were measured in DMF. ^b^E_0−0_ was determined from the intersection of the absorption and emission spectra in DMF.

As shown in Fig. 2a, All sensitizers exhibit two main absorption bands: a high-energy π–π* transition in the 250–350 nm region and a broad, lower-energy band extending through 400–520 nm, which is attributed to intramolecular charge transfer (ICT) from the donor moiety to the electron-deficient pyrimidine acceptor^24^. The bathochromic progression observed from **AS-3** to **AS-1** indicates increasing electron delocalization and stronger donor–acceptor interaction, governed by the donor structure and π-conjugation efficiency. Among all dyes, **AS-1**, which incorporates the strong electron-donating triphenylamine unit, exhibited the most red-shifted and intense ICT band with a maximum absorption at 504 nm and molar extinction coefficient (ε) reached to 4.41 × 10⁴ M^− 1^ cm^− 1^, confirming its superior charge-transfer character. In contrast, **AS-2**, containing an ethyl-carbazole donor, showed an **λ**_**max**_ at 480 nm (ε = 4.14 × 10⁴ M^− 1^ cm^− 1^), while **AS-3**, with a piperonal donor, displayed a more blue-shifted ICT band at 463 nm (ε = 3.16 × 10⁴ M^− 1^ cm^− 1^), consistent with its weaker molecular geometry/conjugation and reduced conjugation. The **AS-4** dye, featuring a pyrene donor, exhibited intermediate absorption behavior at 483 nm (ε = 4.29 × 10⁴ M⁻¹ cm⁻¹), reflecting its rigid planar π-framework that enhances electron delocalization and light absorption in the visible region. The emission spectra (Fig. 2b) of the four sensitizers further corroborate their strong ICT nature, displaying broad fluorescence bands in the 600–700 nm region. The emission maxima (λ_em_) follow the order **AS-1** (691 nm) **> AS-4** (665 nm) **> AS-2** (638 nm) **> AS-3** (601 nm). The corresponding optical band gaps (E_0−0_), estimated from the intersection of the absorption and emission spectra^25^, were calculated as 2.12, 2.26, 2.39, and 2.19 eV for **AS-1**,** AS-2**,** AS-3**, and **AS-4**, respectively. The smaller band gap of **AS-1** reflects its strong donor strength and extended conjugation pathway, enabling more efficient utilization of lower-energy photons. This behavior is advantageous for light harvesting and photocurrent generation in (DSSCs)^26^. Conversely, **AS-3**, with the highest band gap (2.39 eV), absorbs within a narrower spectral range, limiting its contribution to visible-light absorption. The sequence of ICT intensity and spectral red-shift can therefore be expressed as **AS-1 > AS-4 > AS-2 > AS-3**, aligning with the increasing conjugation and donor strength across the series. This spectral complementarity is expected to enhance the total photon-to-current conversion efficiency through synergistic light utilization. The molar extinction coefficients (ε = 3.16–4.41 × 10⁴ M^− 1^ cm^− 1^) of the **AS** dyes are significantly higher than that of **N3** (ε ≈ 1.48 × 10⁴ M⁻¹ cm⁻¹) as shown in Fig.S1 in the supplementary file, indicating superior light-harvesting capacity in the visible range^27^. This enhancement can be primarily attributed to the strong donor–acceptor coupling and extended π-conjugation in the pyrimidine organic systems. Thus, when employed together with **N3**, these co-sensitizers are expected to provide broader spectral response and increased (*Jsc*) compared to single-dye systems.

**Fig. 3:**
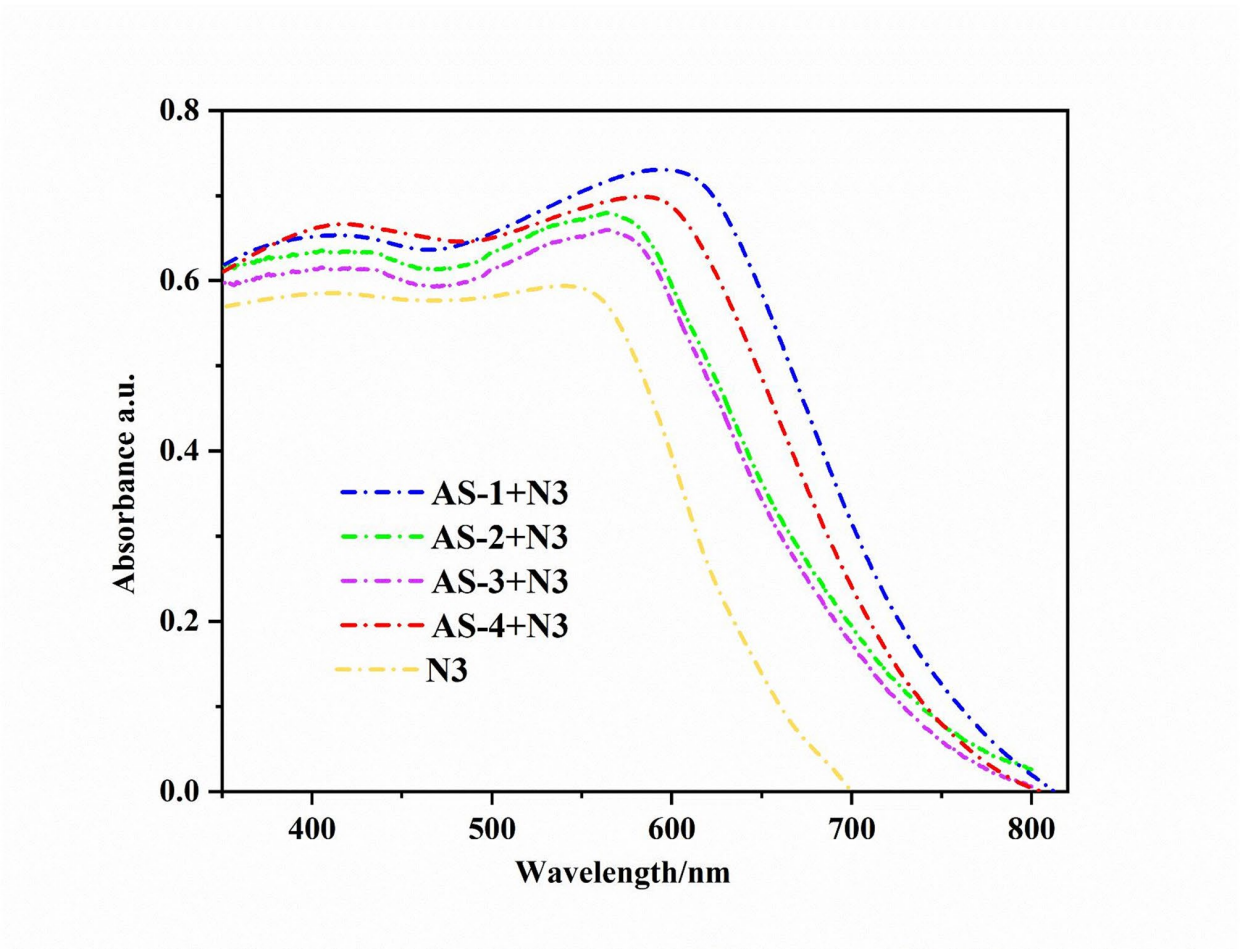
UV–vis absorption spectra of pyrimidine **AS-1-4** sensitizers and **N3** on TiO_2_.

The dye adsorption onto TiO_2_ was markedly improved using the acid–base co-sensitization technique, as confirmed by the UV–Vis absorption spectra presented in Fig. 3. The pyrimidine-based co-sensitizers (**AS-1-AS-4**) showed clear red shifts in their absorption peaks compared to those recorded in solution. These red shifts can be attributed to strong interactions between the carboxylate and pyrimidine nitrogen groups of the sensitizers and the TiO_2_ surface, leading to *J*-type aggregation and deprotonation effects upon adsorption^28–30^. Such spectral changes indicate efficient dye anchoring and electronic coupling between the sensitizer molecules and the semiconductor surface^31^. As shown in Fig. 3, the acid-base co-sensitized systems (**N3 + AS-1–AS-4**) demonstrated wider and more intense absorption bands spanning the 350–700 nm region compared to the single-dye **N3** system. These co-sensitized films exhibited markedly enhanced and broadened absorption features extending toward longer wavelengths, reflecting improved light-harvesting efficiency and greater dye loading on the TiO_2_ surface. The spectral broadening and bathochromic shifts confirm the formation of stable, well-packed dye layers on the TiO_2_ surface through cooperative adsorption of acidic and basic dyes. Among the four pyrimidine co-sensitizers, the **N3 + AS-1** and **N3 + AS-4** systems demonstrated the strongest and widest absorption enhancement, particularly beyond 500 nm, suggesting higher dye-loading efficiency and more extensive visible-light capture. This behavior can be attributed to the strong electron-donating nature of triphenylamine (**AS-1**) and the extended π-system of pyrene (**AS-4**), both of which facilitate effective π–π interactions and promote *J*-type aggregation on TiO_2_. In contrast, the **N3 + AS-3** (piperonal donor) system showed the lowest absorption intensity in the visible region, consistent with its weaker donor strength and limited surface binding. The enhanced optical performance of the acid–base co-sensitized films **(N3 + AS-1-AS-4)** arises from the complementary absorption characteristics of the two dyes and their distinct surface-binding modes^32^. The **N3 dye**, being acidic, anchors primarily through carboxylate groups to Brønsted-acid sites on TiO_2_, while the pyrimidine co-sensitizers interact through their basic nitrogen centers with Lewis-acid sites on the semiconductor surface^33^. This complementary anchoring minimizes competitive dye adsorption, suppresses aggregation, and reduces charge recombination at the TiO_2_/electrolyte interface. This leads to a more homogeneous distribution of dyes and intensified light absorption over a broader wavelength range. The expanded and red-shifted absorption spectra of the acid–base systems (**N3 + AS-1-AS-4**), relative to the single or acid–acid sensitization setups, clearly confirm the efficiency of the acid–base co-sensitization approach in improving both dye adsorption and overall light-harvesting performance. Among the studied systems, **N3 + AS-1** and **N3 + AS-4** achieved the most favorable spectral overlap and optical density, confirming that combining triphenylamine or pyrene donors with the pyrimidine acceptor in the co-sensitization process provides optimal charge-transfer pathways^34^. FT-IR spectra of **AS-1-4** on TiO₂ has been mentioned in supplementary file (**Figures S3**, **S8**, **S13** and **S18**).

### Electrochemical properties

**Fig. 4:**
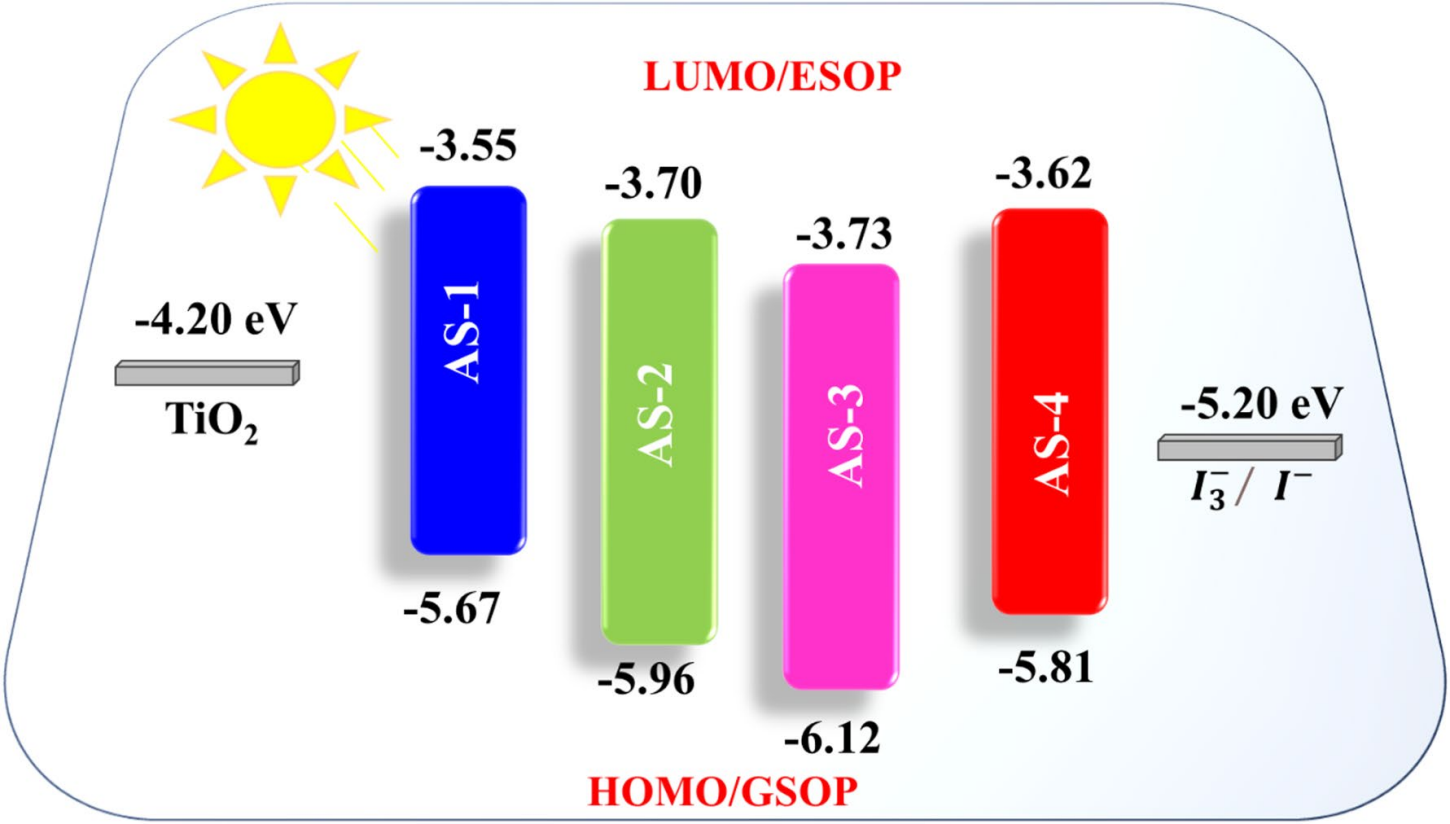
Experimental HOMO-LUMO levels values for pyrimidine co-sensitizer **AS-1-4.**

The electrochemical behavior of the acid–base co-sensitizers **AS-1-AS-4** was examined through cyclic voltammetry (CV), with the corresponding data summarized in Table 2. The energy levels of the highest occupied molecular orbital (HOMO) and the lowest unoccupied molecular orbital (LUMO) are key parameters that determine the electron-donating and electron-accepting characteristics of the dye molecules^35,36^. For **AS-1**, the HOMO energy level was found to be -5.67 eV, while for **AS-2**,** AS-3**, and **AS-4**, the HOMO levels were − 5.96 eV, -6.12 eV, and − 5.81 eV (Fig. 4), respectively. These values lie well below the redox potential of the I^-^/I_2_^-^ electrolyte (-5.20 eV), ensuring sufficient thermodynamic driving force for dye regeneration following photoexcitation^37^. The LUMO energy levels \were calculated using the following equation:

1 $$\:\mathrm{E}\mathrm{S}\mathrm{O}\mathrm{P}=\:-\:\left[\right({\mathrm{E}}_{\mathrm{o}\mathrm{n}\mathrm{s}\mathrm{e}\mathrm{t}}^{\mathrm{o}\mathrm{x}\mathrm{d}}+4.7)-\mathrm{E}_{0-0}]\:\mathrm{e}\mathrm{V}$$

Based on this relation, the LUMO energies were estimated at -3.55 eV **(AS-1)**, -3.70 eV **(AS-2)**, -3.73 eV **(AS-3)**, and − 3.62 eV **(AS-4)** as shown in Fig. 4. These LUMO values are located above the conduction band edge of TiO_2_ (≈ -4.20 eV), confirming the suitability of all dyes for efficient electron injection into TiO_2_, a key requirement for high-performance (DSSCs). A smaller E_0−0_ value corresponds to stronger visible-light absorption and enhanced (ICT), as previously observed in the UV–Vis results. This correlation confirms that donor strength and conjugation length directly influence both the optical and electrochemical properties of the pyrimidine dyes^37^.

**Table 2:** Electrochemical data of **AS-1-4** sensitizers.

Dyes	Experimental						Theoretical		
	E_OX_ (HOMO)	E_ox_^*^ (LUMO)	E_0−0_ (eV)	∆G_inj_ (eV)	∆G_reg_ (eV)	∆G_rec_ (eV)	E_OX_ (HOMO)	E_ox_^*^ (LUMO)	E_0−0_ (eV)
**AS-1**	-5.67	-3.55	2.12	-0.65	0.47	1.47	-5.53	-3.53	2.00
**AS-2**	-5.96	-3.70	2.26	− 0.50	0.76	1.76	-5.91	-3.72	2.19
**AS-3**	-6.12	-3.73	2.39	-0.47	0.92	1.92	-6.17	-3.80	2.37
**AS-4**	-5.81	-3.62	2.19	-0.58	0.61	1.61	-5.79	-3.64	2.15

The free energy of electron injection (ΔG_inj_) was calculated using the equation:

2 $$\:\varDelta\:G_{inj}\:\left(eV\right)\:=\:{E}_{OX}^{{dye}^{*}}-\:{E}_{CB}$$

The ΔG_inj_ values were found to be -0.65 eV (**AS-1**), -0.50 eV (**AS-2**), -0.47 eV (**AS-3**), and − 0.58 eV (**AS-4**). The negative values indicate that electron injection from the excited dye into TiO_2_ is a thermodynamically spontaneous process for all sensitizers. Among them, **AS-1** exhibits the most negative ΔG_inj_ (-0.65 eV), suggesting faster charge transfer kinetics and higher photovoltaic performance. The high injection potential of **AS-1** can be attributed to its strong donor (triphenylamine) and efficient D-π-A charge-transfer framework^38^.

The free energy for dye regeneration (ΔG_reg_) was calculated according to the following equation:

3 $$\:\varDelta\:G_{reg}\:\left(eV\right)\:=\:{E}_{OX}^{dye}-\:{E}_{redox}$$

where (E_redox_) is the redox potential of the I^-^/I_2_^-^ electrolyte (-5.20 eV). The ΔG_reg_ values were 0.47 eV (**AS-1**), 0.76 eV (**AS-2**), 0.92 eV (**AS-3**), and 0.61 eV (**AS-4).** Lower ΔG_reg_ values indicate faster regeneration of the oxidized dye by the electrolyte. Thus, **AS-1** exhibits the most favorable regeneration kinetics, while **AS-3** requires a higher reorganization energy due to its deeper HOMO and weaker donor strength. Efficient regeneration is critical for maintaining a high steady-state photocurrent and minimizing charge accumulation losses.

4 $$\:\varDelta\:G_{rec.}\:\left(eV\right)=\:{E}_{OX}^{dye}-\:{E}_{CB}$$

The obtained values were 1.47 eV (**AS-1**), 1.76 eV (**AS-2**), 1.92 eV (**AS-3**), and 1.61 eV (**AS-4**). A lower ΔG_rec_. value corresponds to a larger energy barrier for back-electron transfer from TiO_2_ to the oxidized dye, effectively suppressing recombination processes. Therefore, **AS-1** and **AS-4**, with lower ΔG_rec_ values, are expected to show lower charge recombination rates and higher open-circuit voltages (*Voc*). Theoretical DFT calculations using the B3LYP/6-31G(d, p) level of theory were conducted to validate the experimental findings^39^. The calculated HOMO-LUMO energies show excellent agreement with experimental data, confirming the reliability of the computational model. The energy alignment of **AS-1–AS-4** with the **N3** dye suggests that these pyrimidine-based dyes are ideal for acid–base co-sensitization on TiO_2_ electrodes. Among all, **AS-1** (triphenylamine donor) exhibits the best balance of energy parameters, combining a strong injection driving force (ΔG_inj_ = -0.65 eV), low regeneration energy (0.47 eV), and moderate recombination barrier (1.47 eV). These features make **AS-1** the most promising co-sensitizer to enhance the photovoltaic response when coupled with **N3**. All four pyrimidine-based sensitizers satisfy the thermodynamic requirements for efficient electron injection and regeneration in DSSC systems. Both experimental and theoretical analyses confirm that the designed pyrimidine dyes possess optimal HOMO-LUMO alignment relative to TiO_2_ and **N3**, supporting their potential as efficient acid–base co-sensitizers.

### Theoretical calculations for acid-base co-sensitizers **AS-1-4**

**Fig. 5:**
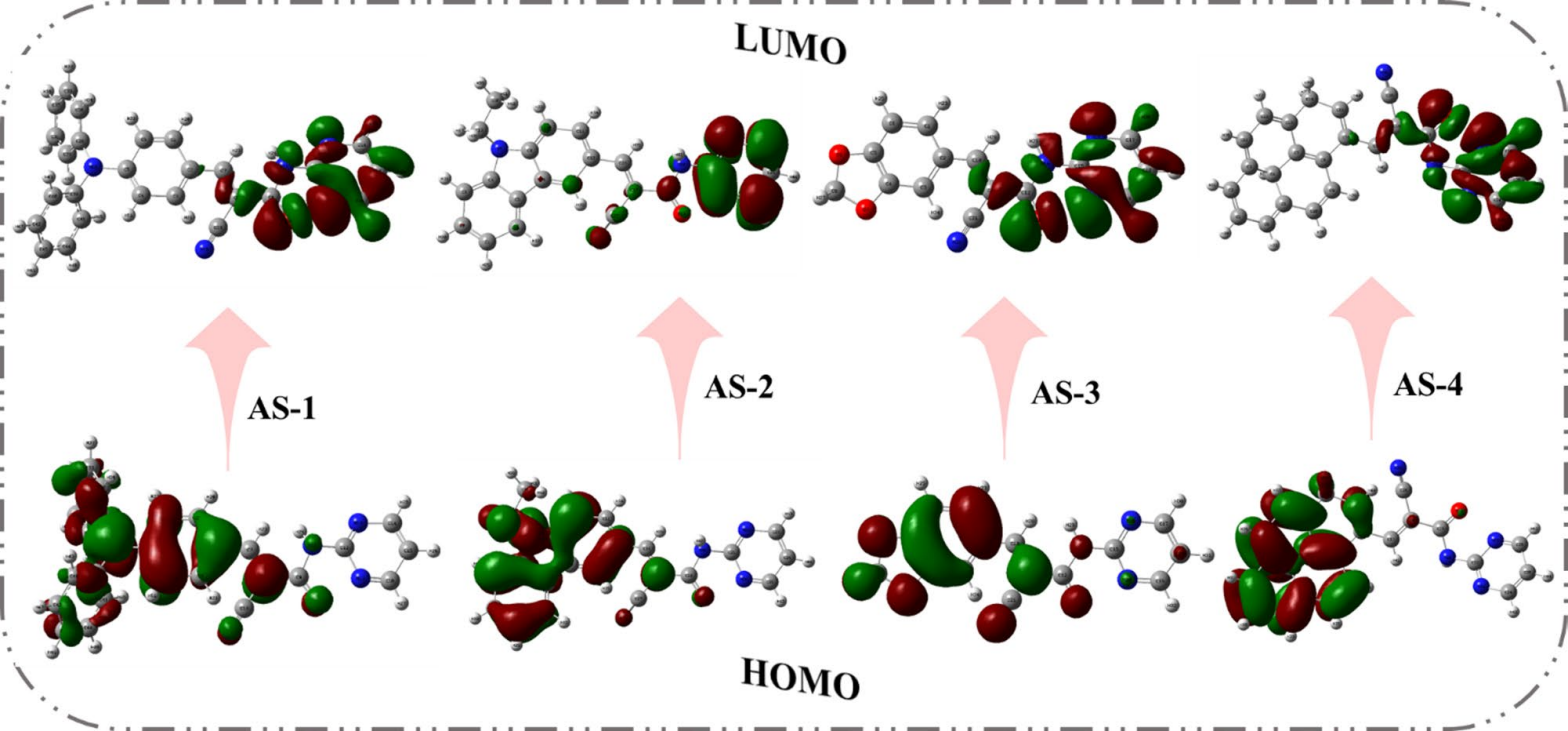
HOMOs and LUMOs geometry for pyrimidine co-sensitizers **AS-1-4**.

The theoretical calculations for acid-base co-sensitizers **AS-1-4** were performed using the B3LYP hybrid functional with the 6-311G(d, p) basis set implemented in the *Gaussian* 09 program^39^. The optimized molecular geometries and corresponding electronic distributions of the (HOMOs) and (LUMOs) are illustrated in Fig. 5. The frontier molecular orbital (FMO) plots clearly reveal a distinct spatial separation of electron density between the HOMO and LUMO levels for all four pyrimidine-based co-sensitizers. In each instance, the HOMO charge density is mainly concentrated on the electron-donating moieties, while the LUMO orbitals are primarily distributed over the pyrimidine acceptor units and the carboxylic anchoring groups^40^. This orbital separation confirms the strong (ICT) nature of the designed D–π–A systems, which facilitates efficient electron migration from the donor to the acceptor upon photoexcitation—an essential requirement for improved photovoltaic performance in DSSCs^41^. For **AS-1**, the HOMO electron density is mainly concentrated on the triphenylamine donor and partially delocalized through the π-conjugated bridge, indicating strong electron-donating ability. The corresponding LUMO is localized over the pyrimidine acceptor and anchoring groups, promoting directional electron injection into the TiO_2_ conduction band. In **AS-2**, the HOMO is confined mostly to the ethyl-carbazole moiety, while the LUMO remains distributed on the pyrimidine. The clear separation between donor-localized HOMO and acceptor-centered LUMO suggests favorable charge-transfer characteristics, though slightly less pronounced than in **AS-1** due to the shorter conjugation path. For **AS-3**, the HOMO is primarily localized on the piperonal donor ring, reflecting its weaker electron-donating character caused by the presence of the substituent. The LUMO density is limited to the pyrimidine acceptor, indicating reduced orbital overlap and weaker ICT efficiency. In **AS-4**, both HOMO and LUMO orbitals show extensive delocalization owing to the planar π-conjugated pyrene structure. The HOMO extends across the pyrene and linker region, while the LUMO resides mainly on the pyrimidine and anchoring groups. This configuration ensures strong π–π coupling and improved charge separation efficiency compared to **AS-2** and **AS-3.**

### Molecular electrostatic potential (MEP) analysis

**Fig. 6:**
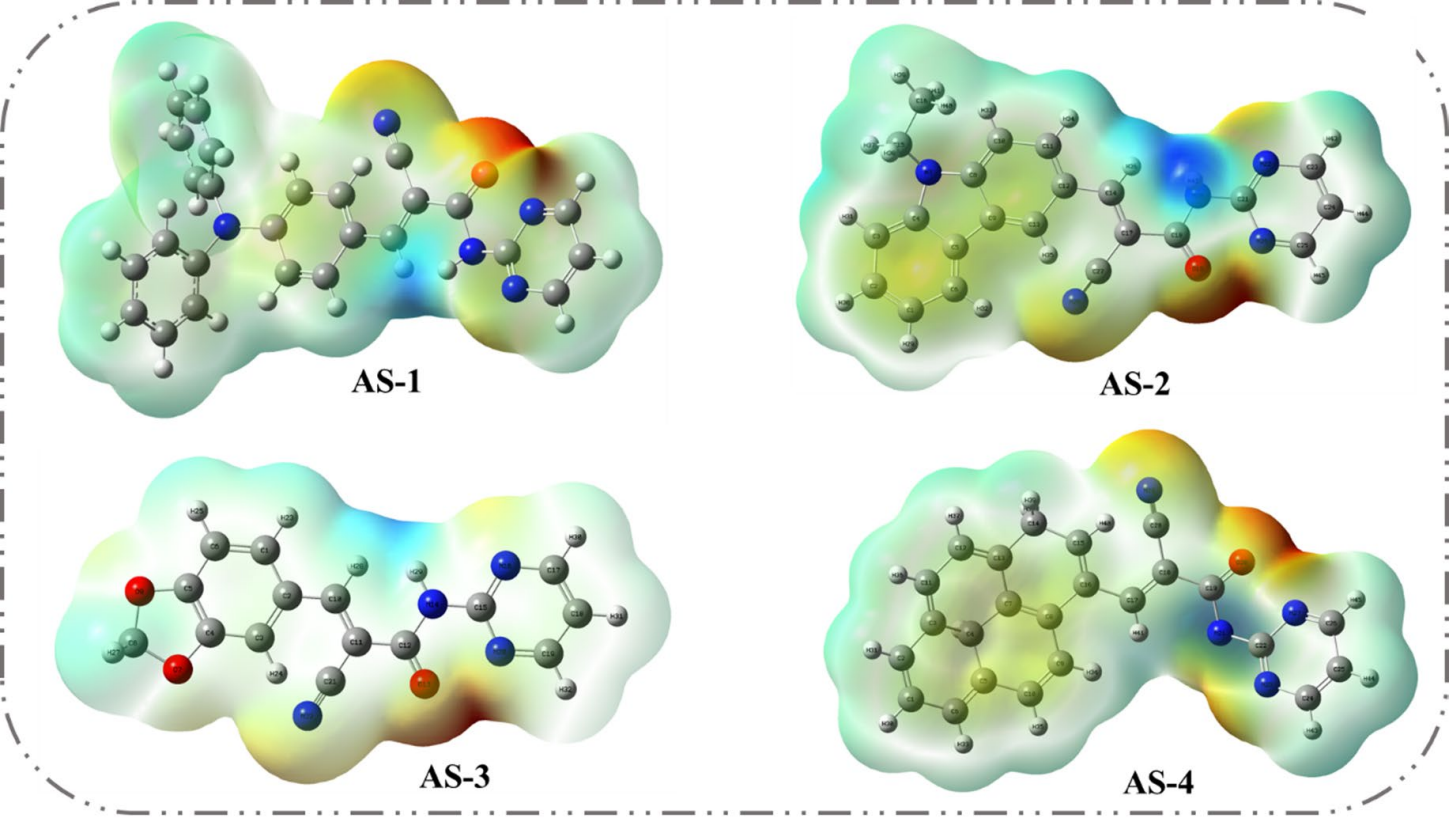
(MEP) of pyrimidine co-sensitizers **AS-1-4.**

Molecular electrostatic potentials (MEPs) are an effective tool for understanding the internal charge-transfer (ICT) characteristics of organic dyes and for visualizing regions of electrophilic and nucleophilic reactivity^42^. In this study, MEP analysis was performed to investigate the charge-distribution patterns of the pyrimidine-based co-sensitizers **AS-1-AS-4**. The influence of different donor groups (triphenylamine, ethyl-carbazole, piperonal, and pyrene) and the common pyrimidine acceptor was assessed by analyzing the electrostatic potential surfaces, as illustrated in Fig. 6. In **AS-1**, the negative (red) potential regions, representing areas of high electron density are mainly concentrated around the anchoring carbonyl, cyano and pyrimidine nitrogen sites. These regions correspond to the electron-accepting domains responsible for binding to TiO_2_ and facilitating electron injections. Conversely, the positive (blue) potential regions, indicative of lower electron density, are located on the triphenylamine donor rings, reflecting their strong electron-donating character and role in initiating ICT toward the pyrimidine acceptor. For **AS-2**, the negative potential is distributed over the pyrimidine core and carbonyl groups, while the positive regions are localized on the ethyl-carbazole donor. This polarity suggests efficient donor-to-acceptor charge transfer through the π-conjugated system. For **AS-4**, the MEP surface reveals pronounced positive potential across the planar pyrene moiety, while the negative regions remain centered on the pyrimidine and carboxyl groups. This distinct charge separation highlights strong donor–acceptor polarization, promoting enhanced electronic coupling and efficient electron injection into TiO_2_. MEP analysis confirms that the pyrimidine co-sensitizers exhibit well-defined electronic polarity, with the negative potential zones corresponding to anchoring and electron-accepting sites, and the positive regions associated with donor activity. The trend in charge-separation efficiency follows **AS-1 > AS-4 > AS-2 > AS-3**, consistent with their optical and electrochemical behavior. These findings provide deeper insight into the electronic distribution and reactivity of the co-sensitizers, explaining their effectiveness in acid–base co-sensitization with **N3**. The complementary charge distribution of the pyrimidine dyes and N3 enhances electron transfer, suppresses recombination, and ultimately improves the light-harvesting performance of dye-sensitized solar cells.

### Photovoltaic characterizations for pyrimidine co-sensitizers AS-1-4

**Fig. 7:**
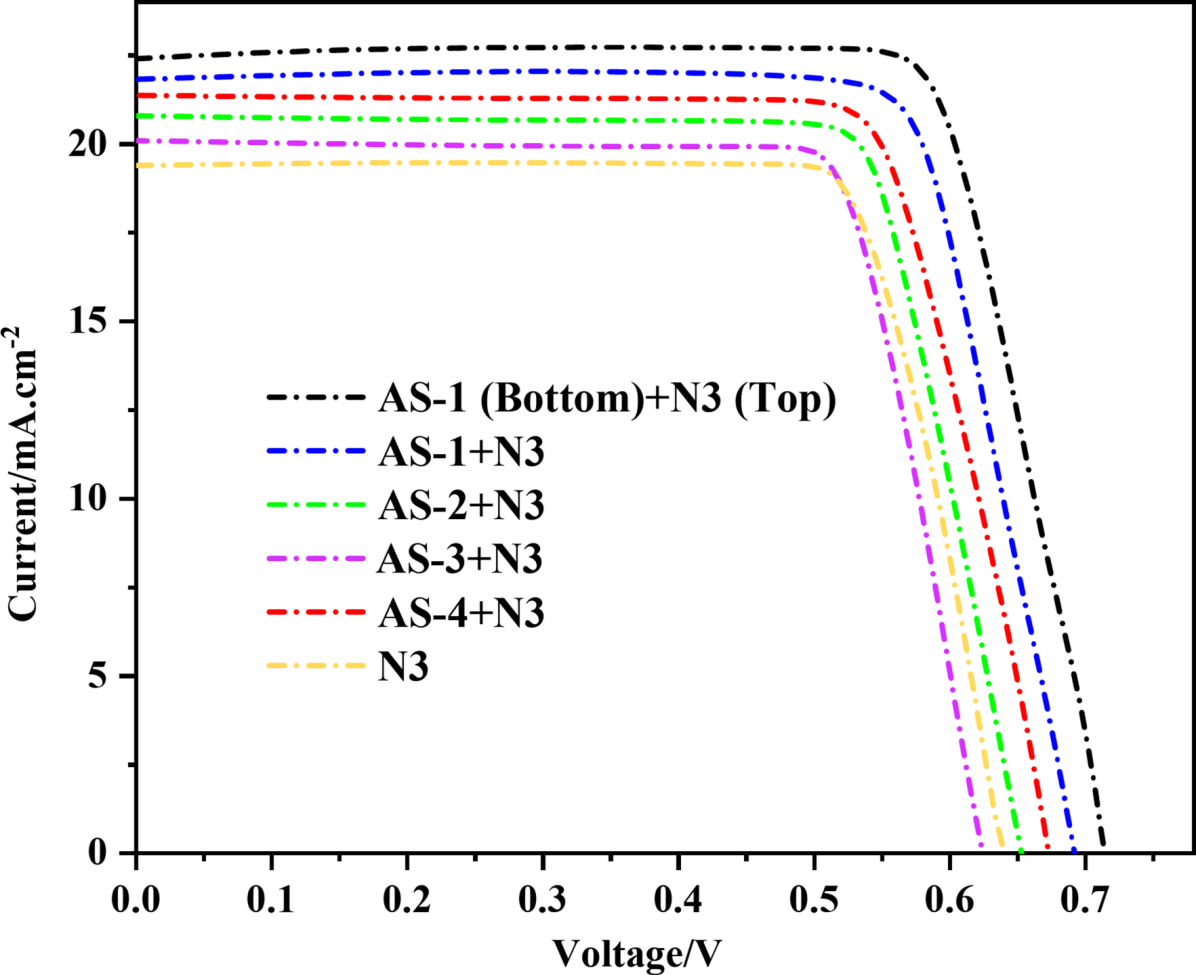
*I-V* curves of pyrimidine acid-base co-sensitizers **AS-1-4** with **N3** and PT-DSSC based on **AS-1 (Bottom)** and **N3 (Top)**.

In this work, the photovoltaic characteristics of DSSCs were evaluated through I-V measurements under standard AM 1.5G illumination, with particular emphasis on assessing the influence of the acid-base co-sensitization strategy, as illustrated in Fig. 7^43^. The well-known Ru-based dye **N3**, an acidic sensitizer with strong carboxyl anchoring groups, was used as a reference system. The pyrimidine-based organic co-sensitizers **AS-1-AS-4**, featuring different donor moieties (triphenylamine, ethyl-carbazole, piperonal, and pyrene, respectively), were employed to explore how donor structure and acid-base interactions influence photovoltaic performance. The measured photovoltaic parameters, including *(V*_*OC*_*)*,* (J*_*SC*_*)*, fill factor *(FF)*, and power conversion efficiency (η_cell_), are summarized in Table 3. Dye loading experiments are commonly used to better realize the effect of several anchors on dye performance. Considering this, a DMF/H_2_O (1:1) combination containing 0.1 M NaOH was used to desorb dye from the TiO_2_ surface in order to quantify the total quantity of dye adsorbed on the TiO_2_. Dye-loading measurements reveal that acid–base co-sensitized and tandem devices exhibit significantly higher surface concentrations of adsorbed dye compared to the single N3 system. The highest dye loading observed for the **AS-1 (Bottom) + N3 (Top)** tandem configuration directly correlates with the enhanced Jsc, confirming that increased dye uptake, enabled by complementary carboxylate and pyrimidine anchoring interactions, plays a key role in the improved photovoltaic performance. The improved photovoltaic performance of the co-sensitized and tandem devices arises from complementary bonding interactions at the TiO₂/dye interface. The **N3** dye anchors strongly to TiO₂ through its carboxylate groups, forming stable bidentate Ti–OOC bonds with surface Ti(IV) sites, which ensure efficient electron injection. In contrast, the pyrimidine fluorescent dyes coordinate through Lewis-basic nitrogen atoms to unsaturated Ti(IV) surface sites (N→Ti), with additional stabilization from hydrogen-bonding interactions with Ti–OH groups. This dual-site adsorption reduces competitive dye binding, enhances overall dye loading, and improves interfacial charge separation. As a result, higher photocurrent densities are achieved in the acid-base co-sensitized and tandem configurations^43,44^.

**Table 3:** Photovoltaic parameters for pyrimidine acid-base co-sensitizer **AS-1-4**, and PT-DSSC based on **N3**, and **AS-1** measured at an irradiation of 100 mW.cm^− 2^ and simulated AM 1.5 sunlight.

Sensitizers	Device type	V_OC_^a^ (V_OC_^b^)/mV	J_SC_^a^ (J_SC_^b^) (mA.cm^− 2^)	FF^a^ (FF^b^)/%	PCE^a^ (PCE^b^)/%	Concentration of the dye/10^− 5^ mol cm^− 2^
(N3)	Co-sensitization	641 (640 ± 2.80)	19.35 (19.37 ± 0.0524)	53.21	6.60 (6.66 ± 0.05)	2.32
(AS-1 + N3)	Acid-base co-sensitization	694 (693 ± 3.71)	21.90 (21.96 ± 0.07)	63.03	9.58 (9.54 ± 0.08)	3.22
(AS-2 + N3)	Acid-base co-sensitization	654 (655 ± 2.8)	20.83 (20.82 ± 0.08)	62.53	8.51 (8.50 ± 0.02)	2.98
(AS-3 + N3)	Acid-base co-sensitization	627 (628 ± 1.08)	20.09 (20.09 ± 0.10)	61.45	7.74 (7.72 ± 0.06)	2.72
(AS-4 + N3)	Acid-base co-sensitization	676 (676 ± 3.66)	21.45 (21.40 ± 0.07)	62.75	9.10 (9.11 ± 0.07)	3.10
(AS-1 (Bottom)+ N3 (Top))	P-Tandem	717 (717 ± 2.08)	22.52 (22.49 ± 0.06)	68.86	11.12 (11.10 ± 0.03)	3.45

^a^The best device parameters (listed in the manuscript), ^b^The average device parameters (obtained from three devices).

The single-dye **N3** cell achieved a *V*_*OC*_ of 641 mV, *J*_*SC*_ of 19.35 mA·cm^− 2^, *FF* of 53.21**%**, and an overall efficiency of 6.60%. This performance is consistent with previously reported values for **N3**-based DSSCs, which show strong absorption in the red region but limited response in the blue–green part of the solar spectrum^43^. When co-sensitized with the pyrimidine dyes, a significant enhancement in photovoltaic efficiency was observed for all systems, demonstrating the effectiveness of the acid–base co-sensitization technique. Among the studied acid-base co-sensitizers, the **AS-1 + N3** device exhibited the highest efficiency of 9.58%, with *V*_*OC*_ = 694 mV, *J*_*SC*_ = 21.90 mA·cm^− 2^, and *FF* = 63.03%, corresponding to an increase of approximately 45% compared to the **N3**-only cell. This improvement is attributed to the strong electron-donating nature of the triphenylamine group in **AS-1**, which promotes efficient charge transfer toward the pyrimidine acceptor and improves interfacial electron injection into TiO_2_. The acid–base co-adsorption between the basic pyrimidine nitrogen of **AS-1** and the acidic carboxylate groups of **N3** results in more uniform dye loading, suppressed aggregation, and minimized back-electron recombination, all contributing to the higher ***J***_***SC***_ and ***V***_***OC***_^44^. The acid-base co-sensitizers, **AS-4 + N3** combination achieved 9.10**%** efficiency, corresponding to a 38% improvement over **N3** alone. This enhancement is primarily due to the planar π-conjugated pyrene donor, which provides strong light absorption and superior charge delocalization. The acid-base co-sensitizers, **AS-2 + N3** co-sensitized cell also performed well, with 8.51% efficiency a 29% increase over **N3**, benefiting from the balanced donor strength and structural rigidity of the ethyl-carbazole moiety. In contrast, acid-base co-sensitizers, **AS-3 + N3**, incorporating the piperonal donor, produced a lower efficiency of 7.74%, which represents a modest 17% improvement compared with **N3**. This lower enhancement is attributed to the weak donor ability and limited ICT strength of the piperonal unit, consistent with its higher energy gap and smaller electron-injection driving force observed in electrochemical studies. The most remarkable enhancement was achieved in the tandem layered configuration **(AS-1 (bottom) + N3 (top)**, which delivered a *V*_*OC*_ of 717 mV, *J*_*SC*_ of 22.52 mA·cm^− 2^, *FF* of 68.86%, and an impressive overall efficiency of 11.12%. This corresponds to a 68.5% increase in cell efficiency compared to the **N3-**only system^45^. The outstanding performance of this tandem configuration arises from the optimized molecular orientation and complementary spectral response of the two dyes. The bottom **AS-1 layer** efficiently injects electrons into TiO_2_ through its pyrimidine anchoring, while the top **N3 layer** extends light absorption into the red–NIR region and protects the TiO_2_ surface from direct electrolyte contact. This arrangement enhances the open-circuit voltage through dipole-induced surface potential effects and improves both J_SC_ and FF by promoting directional charge transfer and suppressing recombination^46^. These results confirm that the acid–base co-sensitization mechanism effectively enhances interfacial charge transfer and dye coverage by exploiting complementary anchoring interactions^46^.

### Incident photon-to-current conversion efficiency (IPCE) analysis for AS-1-4

**Fig. 8:**
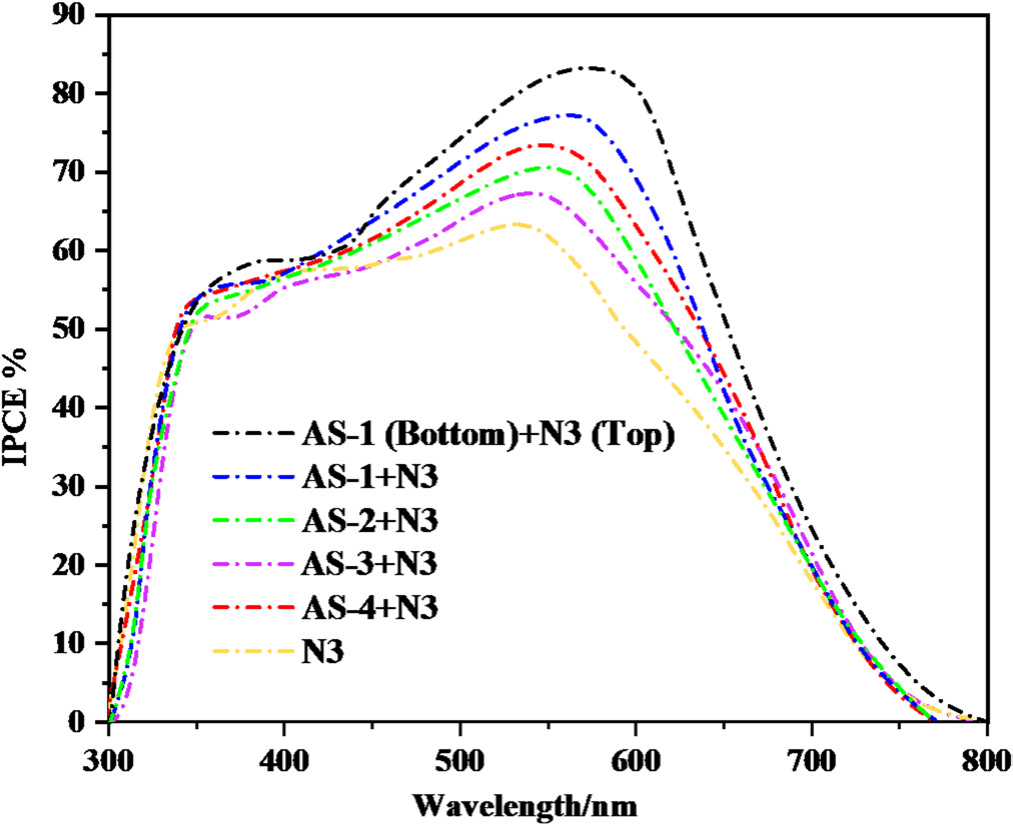
*IPCE* curves of pyrimidine acid-base co-sensitizers **AS-1-4** and **N3** and PT-DSSC based on **AS-1-3** and **N3**.

The Incident Photon-to-Current Conversion Efficiency *IPCE* spectra presented in Fig. 8 represents the wavelength-dependent light-harvesting efficiencies of DSSCs sensitized with the pyrimidine-based co-sensitizers **AS-1-AS-4** and the Ru-based **N3 dye and** PT-DSSC based on **N3**, and **AS-1** measured at an irradiation of 100 mW.cm^− 2^ and simulated AM 1.5 sunlight^47^. Each co-sensitization system was designed to explore the role of acid–base interactions between the pyrimidine moiety (basic site) and the carboxyl groups of **N3** (acidic site) in enhancing spectral response and photocurrent generation. The reference **N3-**sensitized device exhibited a peak *IPCE* of approximately 65% around 530 nm, corresponding to the characteristic metal-to-ligand charge transfer (MLCT) transition of Ru-based complexes. However, its *IPCE* intensity declined sharply in the blue region (below 450 nm), indicating a limited absorption window. When co-sensitized with the pyrimidine-based dyes, the *IPCE* spectra revealed broader and more intense responses across the 350–700 nm range, demonstrating the effectiveness of the acid–base co-sensitization strategy in expanding the light-harvesting capability^48^. The acid-base co-sensitized **AS-1 + N3** system achieved a marked improvement in light-harvesting ability, showing a peak *IPCE* of approximately 77%. This enhancement can be attributed to the synergistic interaction between **AS-**1’s triphenylamine donor, which strongly absorbs in the blue–green region, and **N3’s** broader absorption that extends toward the red region. The complementary absorption and efficient electronic coupling between the two dyes result in a stronger and wider *IPCE* response, consistent with the higher J_SC_ (21.90 mA·cm^− 2^) and overall efficiency (9.58%) observed in the *I–V* characteristics. Acid–base co-sensitized (**AS-4 + N3)** combination also demonstrated efficient photon capture, achieving a peak *IPCE* value of approximately 73%. The extended π-conjugation and planarity of the pyrene donor in **AS-4** facilitate stronger π–π interactions with TiO_2_, improving both charge delocalization and light absorption. Similarly, the acid–base co-sensitized **AS-2 + N3** system exhibited a moderate *IPCE* response (~ 70%) due to the ethyl-carbazole donor, which maintains a good balance between spectral overlap and charge transfer. The acid-base co-sensitized **AS-3 + N3** device, while showing a broader absorption than N3 alone, reached a comparatively lower *IPCE* of 67%, consistent with its weaker donor strength and reduced charge-separation efficiency. For Pt-tandem of **(AS-1 bottom + N3 top)** displayed the highest light-harvesting efficiency, with *IPCE* values exceeding 83% and a broad spectral response extending up to 720 nm. The outstanding performance of this tandem configuration can be explained by the complementary absorption of **AS-1** and **N3**, which together harvest photons efficiently across the full visible spectrum. The bottom **AS-1** layer injects electrons rapidly into the TiO_2_ conduction band through its pyrimidine anchoring sites, while the upper **N3 layer** absorbs longer wavelengths and reduces recombination by forming a protective molecular barrier at the TiO_2_/electrolyte interface. The broader and more intense *IPCE* spectra observed for Tandem **AS-1 (bottom) + N3 (top)**, clearly demonstrate improved photon utilization, efficient electron injection, and reduced interfacial charge recombination^49^. All the details for the tandem configuration has been shown in **Fig.S22** and mentioned in the supplementary file.

**Fig. 9:**
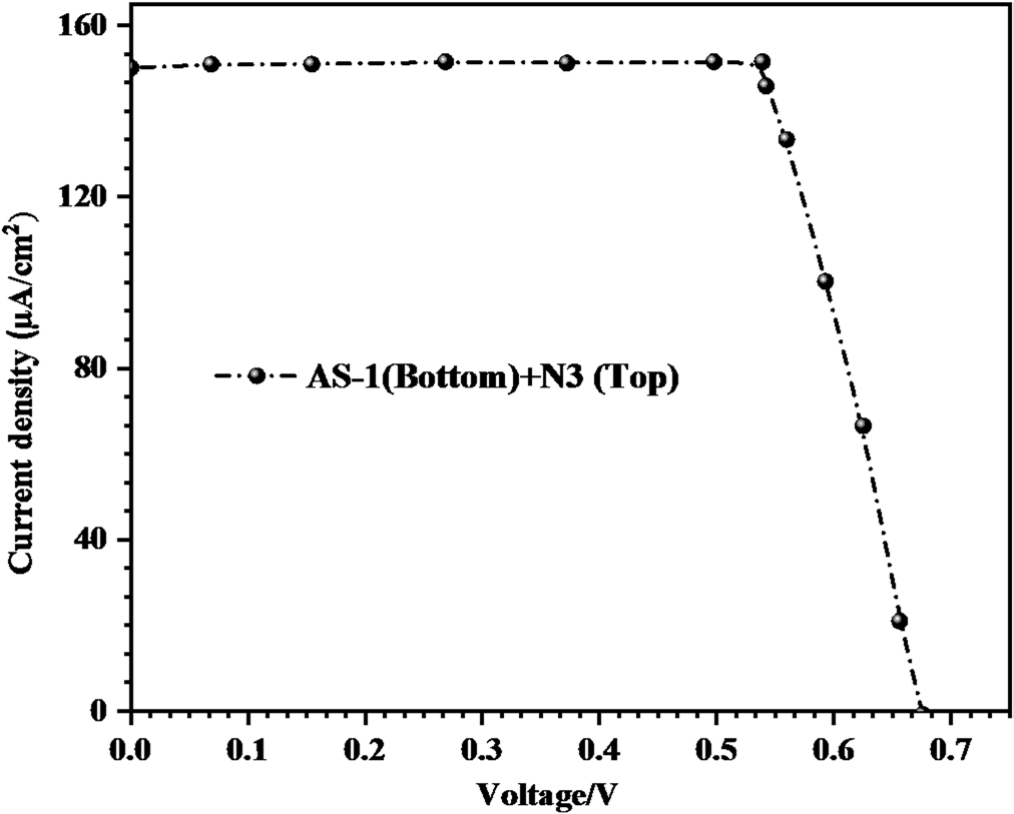
*J*-*V* curve under 1000 lx illumination for Tandem **AS-1** and **N3.**

To further evaluate the light-harvesting capability and charge-transfer behavior of the developed dyes under low-intensity illumination, *J-V* measurements were performed at 1000 lx (0.283 mW cm⁻²) for the tandem configuration **(N3 top + AS-1 bottom)**. The corresponding photovoltaic parameters, (*V*_*OC*_), *(J*_*SC*_*)*, fill factor *(FF)*, and power-conversion efficiency (η_cell_) are summarized in Table 4 and shown in Fig. 9.

**Table 4:** *J–V* parameters at 1000 lx (0. 283 mW.cm^− 2^) for tandem **(N3 (top) + AS-1 (bottom)).**

Sensitizer (0.2mM)	Device type	V_OC_^a^ (V_OC_^b^)/mV	J_SC_^a^ (J_SC_^b^) (µA.cm^− 2^)	FF^a^ (FF^b^)/%	PCE^a^ (PCE^b^)/%
N3 (Top) + AS-1 (Bottom)	P-Tandem	678 (678 ± 4.16)	149.50 (149.88 ± 1.293)	61.50 (61.61 ± 0.30)	22.02 (22.34 ± 0.81)

^a^The best device parameters (listed in the manuscript), ^b^The average device parameters (obtained from three devices).

The tandem DSSC exhibited excellent indoor photovoltaic performance, achieving a *V*_*OC*_ of 678 mV, *J*_*SC*_ of 149.50 µA cm^− 2^, *FF* of 61.50%, and an overall efficiency of 22.02% under 1000 lx illumination. This impressive result highlights the strong potential of pyrimidine-based tandem sensitization for indoor energy-harvesting applications, where the spectral power distribution is lower and more concentrated in the visible region compared to standard solar illumination^50^. The **AS-1** dye, with its strong triphenylamine donor and pyrimidine acceptor, forms the bottom layer directly adsorbed onto the TiO_2_ surface. This arrangement facilitates efficient electron injections through the pyrimidine anchoring sites, ensuring strong interfacial coupling between the dye and the semiconductor. The performance improvement demonstrates that charge-transport and recombination dynamics remain efficient even under reduced photon input, indicating strong dye–semiconductor coupling and minimal energy loss pathways^51,52^. The higher efficiency of the layered tandem configuration **(N3 (top) + AS-1 (bottom))** compared to conventional acid–base co-sensitized systems **(AS-1–4 + N3)** arises from the controlled sequential adsorption and spatial separation of the two dyes on a single TiO₂ photoanode. In this configuration, **AS-1** is first anchored directly to the TiO₂ surface, ensuring strong electronic coupling and efficient electron injection through its donor–π–acceptor framework and pyrimidine-based Lewis-basic anchoring. Subsequent adsorption of **N3** as the top layer broadens light absorption toward longer wavelengths while simultaneously passivating the TiO₂ surface and limiting direct electrolyte contact. This ordered bottom/top architecture suppresses competitive adsorption and dye aggregation, increases effective dye loading, and reduces interfacial recombination. Consequently, simultaneous improvements in *Jsc*, *Voc*, and fill factor are achieved, leading to a markedly higher power-conversion efficiency compared to mixed acid–base co-sensitized devices.

### Electrochemical impedance characterization

**Fig. 10:**
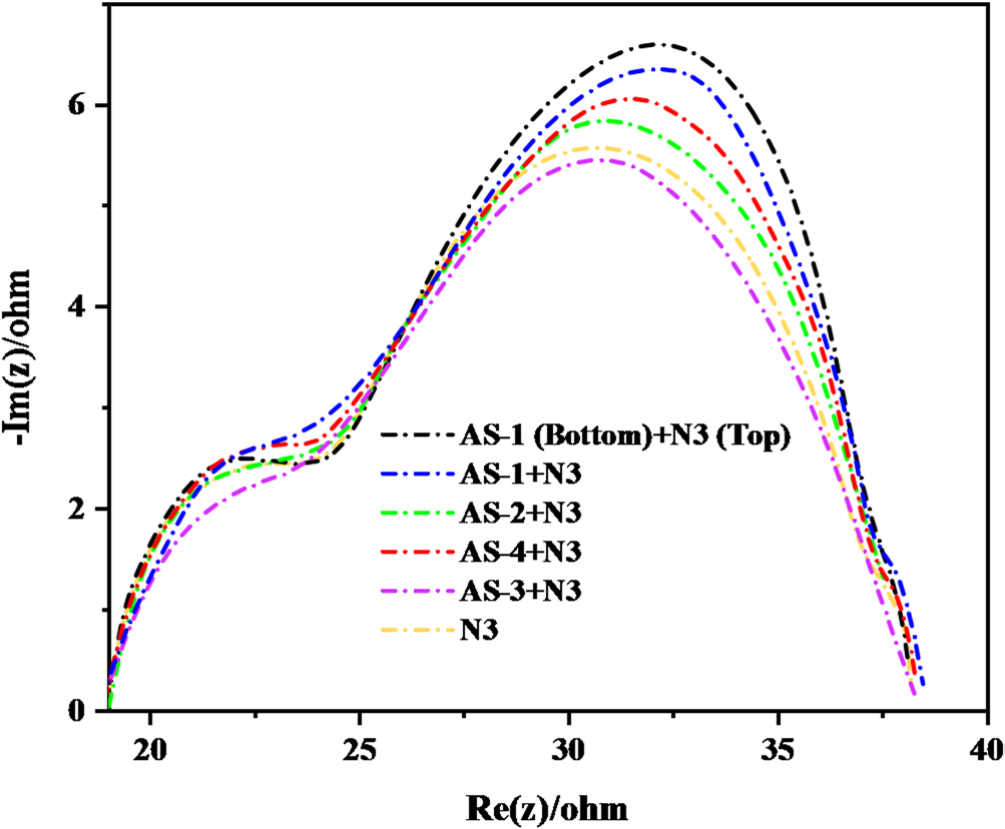
EIS Nyquist plots for pyrimidine co-sensitizers **AS-1-4** and **N3 dye** and PT-DSSC based on **AS-1-3** and **N3**.

Electrochemical Impedance Spectroscopy (EIS) analysis was conducted to investigate the charge-transport and recombination dynamics of the fabricated DSSCs sensitized with pyrimidine-based co-sensitizers (**AS-1-4**) and the Ru-based **N3** dye and **PT-DSSC** based on **N3**, and **AS-1** measured at an irradiation of 100 mW.cm^− 2^ and simulated AM 1.5 sunlight. The Nyquist plots presented in Fig. 10 exhibit two well-defined semicircles, characteristic of dye-sensitized solar cell systems, each corresponding to distinct interfacial processes within the device^53^. The smaller semicircle in the high-frequency region is attributed to the charge-transfer resistance **(R**_**ct**_**)** at the Pt/electrolyte interface, whereas the larger semicircle in the middle-frequency region represents the recombination resistance **(R**_**rec**_**)** occurring at the TiO_2_/dye/electrolyte interface. The diameter of the second semicircle is directly related to the suppression of interfacial charge recombination, which plays a critical role in determining *(V*_*OC*_*)* and overall device efficiency. The EIS spectra reveal that all co-sensitized DSSCs incorporating pyrimidine-based dyes exhibit larger R_rec_ values compared to the single-sensitized **N3** device, indicating more effective inhibition of electron recombination between the injected electrons in TiO_2_ and the oxidized species in the electrolyte^54^. Among the investigated systems, the recombination resistance follows the order: **(N3 (top) + AS-1 (bottom))** > **(AS-1 + N3) > (AS-4 + N3) > (AS-2 + N3) > N3 > (AS-3 + N3)**, demonstrating that **AS-1**, containing a strong triphenylamine donor, provides the most effective charge separation and electron stabilization at the TiO_2_ interface. The increased *R*_*rec*_ for the **AS-1 + N3** and **AS-4 + N3** systems can be attributed to the extended π-conjugation and the presence of electron-rich donor groups, which enhance dye adsorption and strengthen electronic coupling between the dye and TiO_2_. The introduction of the basic pyrimidine moiety in the AS dyes facilitates acid–base interactions with the carboxyl groups of **N3**, leading to improved molecular packing and more uniform surface coverage. This cooperative binding minimizes the formation of charge-recombination sites and prolongs electron lifetime, consistent with the higher V_OC_ and J_SC_ values observed in the photovoltaic data^55^. The higher efficiency of the layered tandem configuration **(N3 (top) + AS-1 (bottom))** compared to conventional acid–base co-sensitized systems (AS-1–4 + N3) arises from the controlled sequential adsorption and spatial separation of the two dyes on a single TiO₂ photoanode. Voc in DSSCs is primarily governed by the position of the TiO₂ quasi-Fermi level and the rate of interfacial recombination at the TiO₂/dye/electrolyte interface. Since the energetic alignment between the dye HOMO–LUMO levels and the I⁻/I₃⁻ redox couple is similar for all investigated systems, differences in Voc are not dominated by energetic offsets. Instead, the enhanced Voc observed for the co-sensitized and tandem devices arises mainly from suppressed recombination and improved surface passivation of TiO₂, as confirmed by increased recombination resistance in EIS measurements. Such behavior reflects reduced intrinsic and extrinsic surface defect activity and more effective interfacial charge separation, consistent with previous working-electrode studies on DSSCs^56^.

**Fig. 11:**
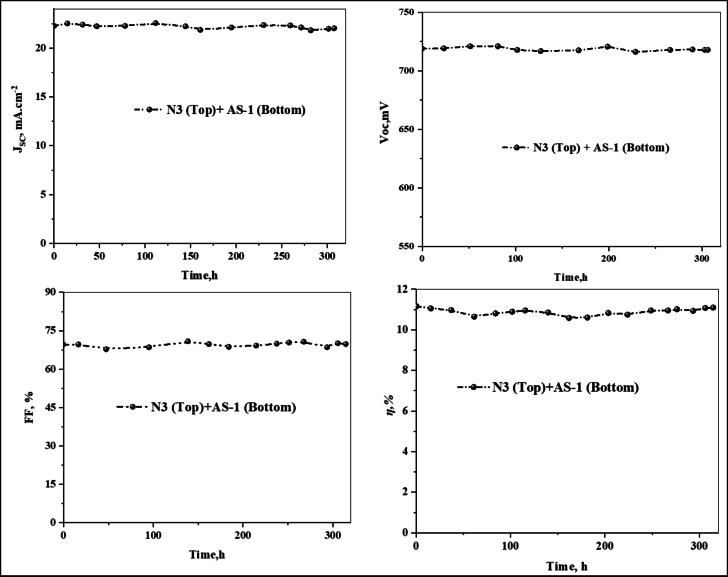
Stability of PT-DSSC based on **AS-1-3** and **N3** under illumination for 300 h.

The long-term operational stability of the fabricated DSSCs was assessed by monitoring the photovoltaic parameters (*V*_*OC*_), (*J*_*SC*_), (*FF*), and (η_cell_), under continuous illumination for 300 h, as shown in Fig. 11 PT-DSSC based on **N3**, and **AS-1** measured at an irradiation of 100 mW cm^− 2^ and simulated AM 1.5 sunlight. The stability tests were conducted for tandem-sensitized **(N3 + AS-1)** system to evaluate the influence on the device’s durability and photostability^56^. The results clearly demonstrate that the **(N3 (top) + AS-1 (bottom))** DSSC exhibited superior long-term stability compared to the single N3-based cell. After 300 h of continuous light exposure, the **(N3 (top) + AS-1 (bottom))** device retained more than 92% of its initial efficiency. This enhanced stability is attributed to the strong dye–TiO_2_ binding provided by the pyrimidine anchoring in **AS-1**, which improves chemical adhesion and minimizes dye desorption over time. The enhanced stability of the **(N3 (top) + AS-1 (bottom))** tandem DSSC, shown in Fig. 11, is attributed to the synergistic molecular interactions and complementary light absorption of the acid–base tandem system. The triphenylamine donor, in **AS-1** contributes to oxidative stability through effective charge delocalization, protecting the dye molecules from photodegradation. Its extended π-conjugation and efficient (ICT) promote uniform charge distribution and minimize local charge accumulation that can accelerate degradation^57^.

## Conclusions

In this work, an acid-base tandem co-sensitization approach was effectively implemented by combining fluorescent pyrimidine-based co-sensitizers (**AS-1-AS-4**) with the Ru(II)-based acidic dye **N3** to fabricate high-performance and durable DSSCs. This selective binding mechanism effectively suppresses competitive dye adsorption, enhances overall surface coverage, and significantly mitigates electron recombination losses, leading to improved interfacial charge transfer and higher (*V*_*OC*_). Among the synthesized pyrimidine sensitizers, **AS-1**, containing a strong triphenylamine donor, exhibited the most favorable optical and electrochemical characteristics. When combined with **N3** in a tandem configuration (**AS-1 bottom + N3 top**), the device achieved an outstanding power conversion efficiency (PCE) of 11.12% under AM 1.5G illumination, representing a 68% enhancement over the single **N3**-based DSSC (6.60%). Under indoor lighting (1000 lx, 0.283 mW cm^-2^), the same tandem cell demonstrated an impressive efficiency of 22.02%, confirming its excellent adaptability to low-light conditions. These results were further supported by **EIS** and IPCE analyses, which revealed higher recombination resistance, prolonged electron lifetimes, and superior light-harvesting efficiency across a broad spectral range. Furthermore, the **(N3 + AS-1)** co-sensitized system displayed excellent long-term stability, retaining over 92% of its initial efficiency after 300 h of continuous illumination. This durability arises from the strong dye–TiO_2_ binding, extended π-conjugation, and balanced charge-transfer dynamics within the tandem structure.

## Supplementary Information

Below is the link to the electronic supplementary material.

Supplementary Material 1

## Data Availability

All data generated or analyzed during this study are included in this published article and its supplementary information files.
