# Supplementary material for: A synergistic acid–base tandem co-sensitization approach using pyrimidine fluorescent dyes achieves 22% indoor efficiency
Source: Sci Rep. 2026 Mar 23;16:9806. doi: 10.1038/s41598-026-40785-z (PMC13018274; doi:10.1038/s41598-026-40785-z)
Supplement: Supplementary file 1 — Supplementary Material 1 [file 41598_2026_40785_MOESM1_ESM.docx]

*Materials*

Alfa Aesar and Sigma Aldrich supplied the starting materials, which. The melting points were measured in degrees Celsius using a Gallenkamp electric melting point instrument and are uncorrected. Thermo Scientific Nicolet iS10 FTIR spectrometer was used to get IR spectra (KBr), while a Burker NMR spectrometer was used to obtain NMR spectra in DMSO-*d6* at frequencies of 400 MHz (^1^H-NMR) and 100 MHz (^13^C- NMR). A high-performance twin beam spectrophotometer (T80 series) was used to acquire UV-visible spectra. Thermo Fisher Scientific DSQ II GC/MS with Kratos MS-70 ev was used for mass analyses, while a Perkin Elmer 2400 analyzer was used for elemental analysis. The attached information file contains detailed information on the instruments and DSSC fabrications used.

2. *Analytical Measurements*


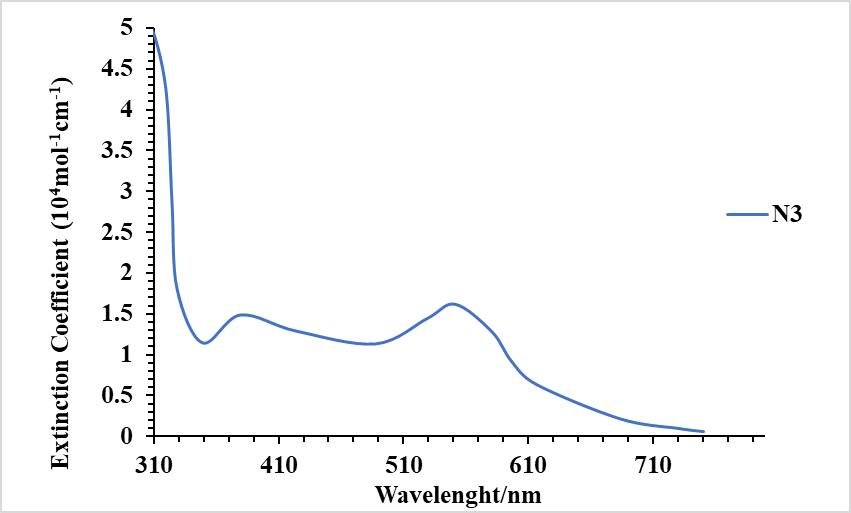


**Figure (S1): UV-Vis. absorption of N3.**


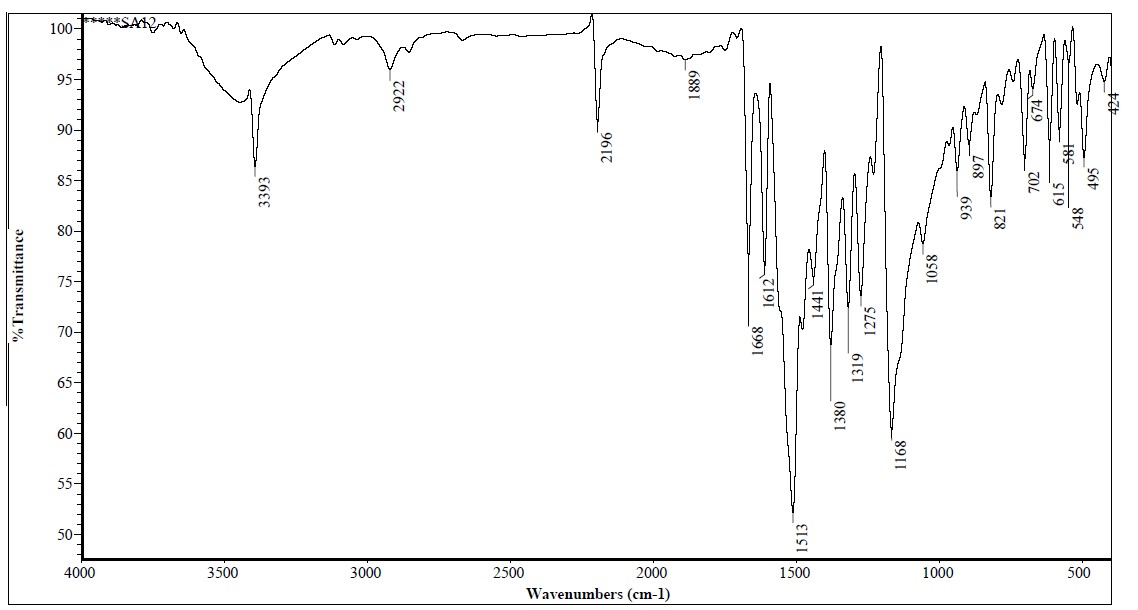


**Figure (S2): IR spectrum of compound AS-1.**


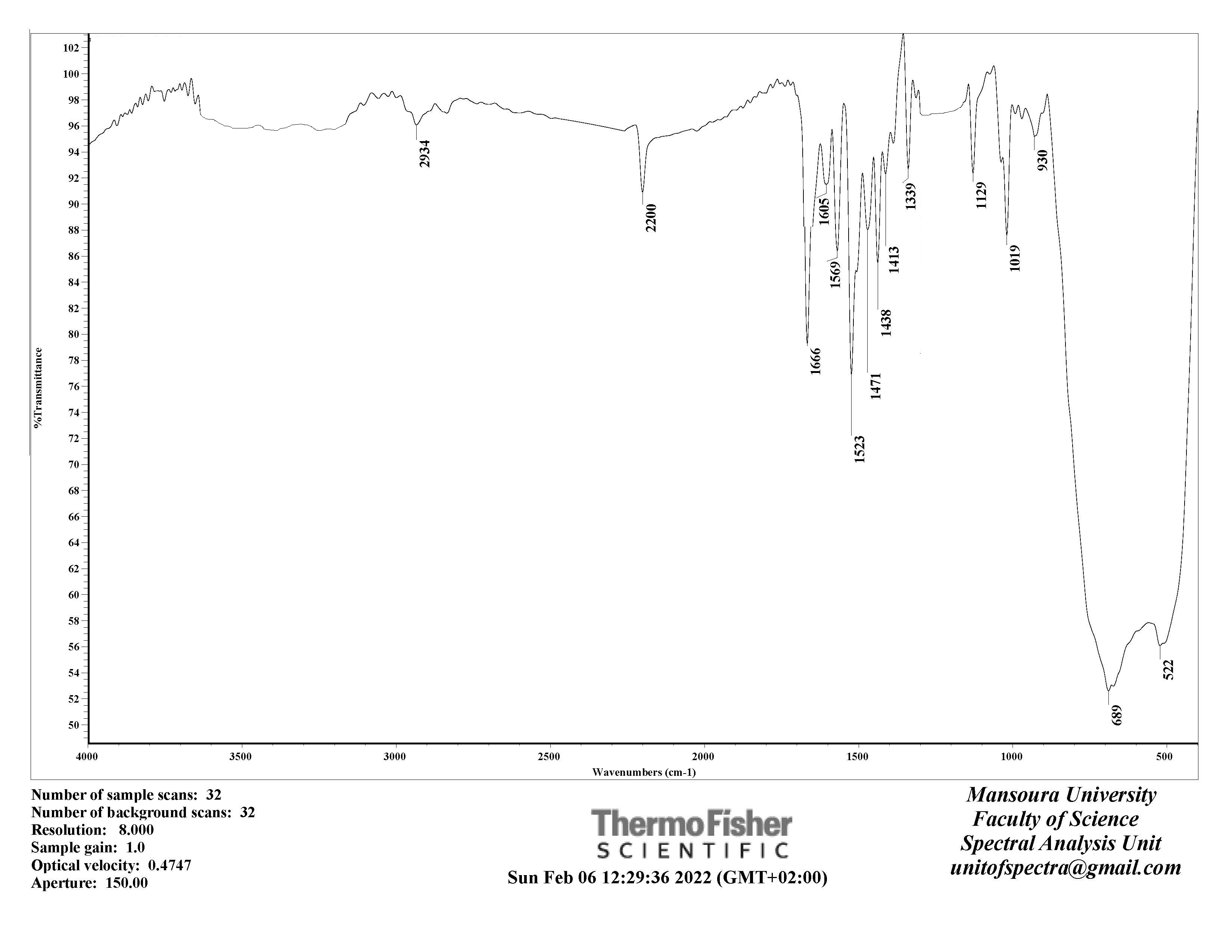


**Figure (S3): IR spectrum of compound AS-1 on TiO_2_.**


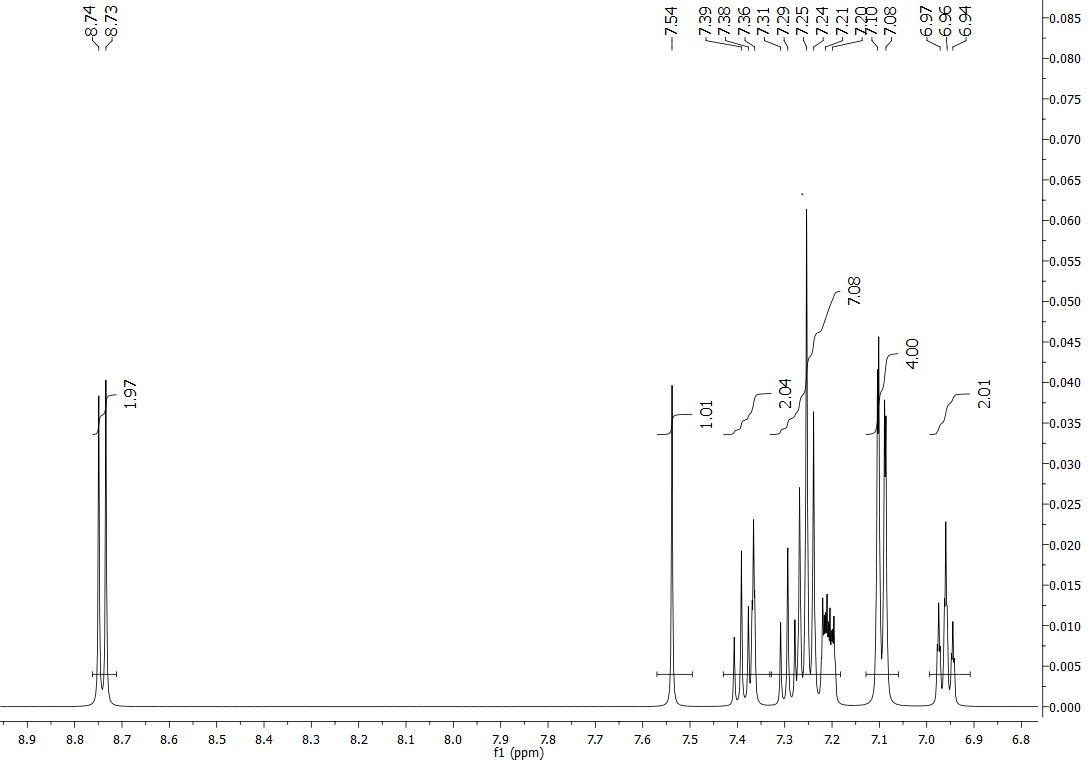


**Figure (S4): ^1^H NMR spectrum of AS-1.**


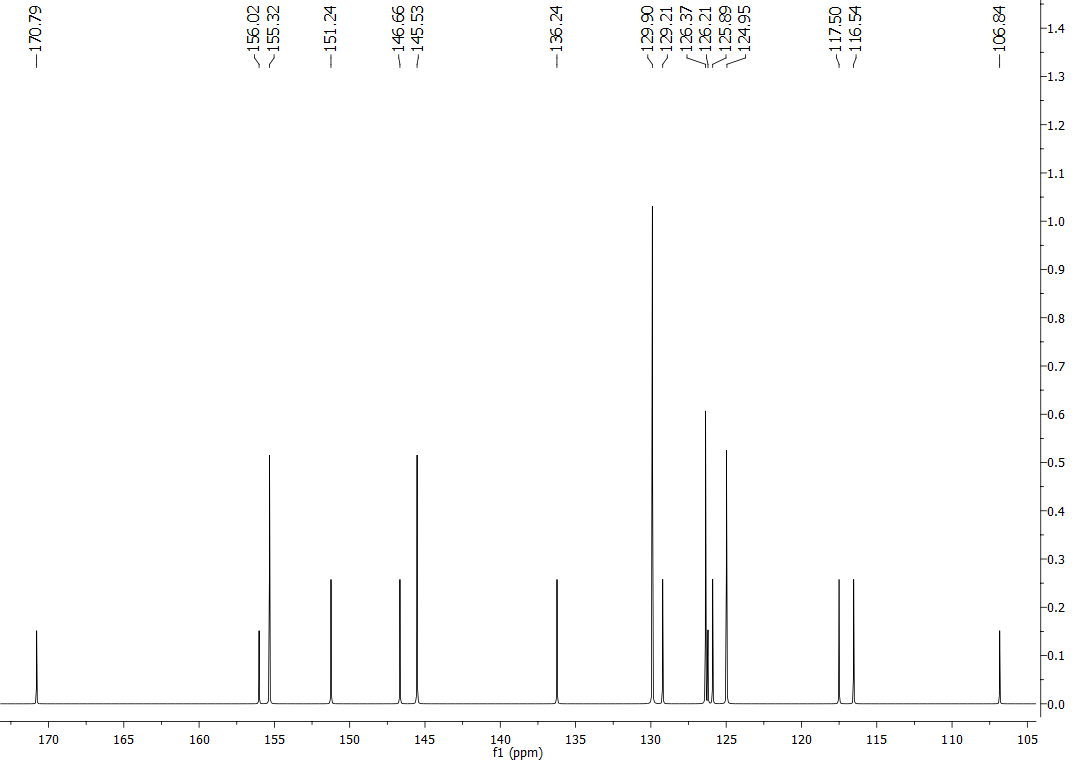


**Figure (S5): ^13^CNMR spectrum of AS-1.**

**Figure S6: Mass spectrum of compound AS-1.**


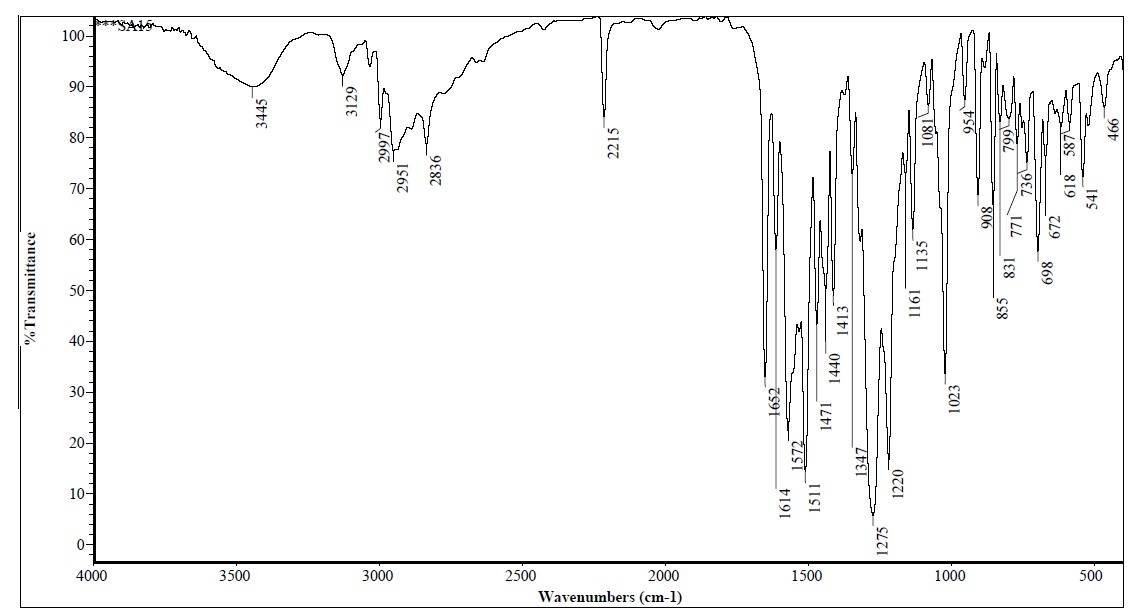


**Figure (S7): IR spectrum of compound AS-2.**


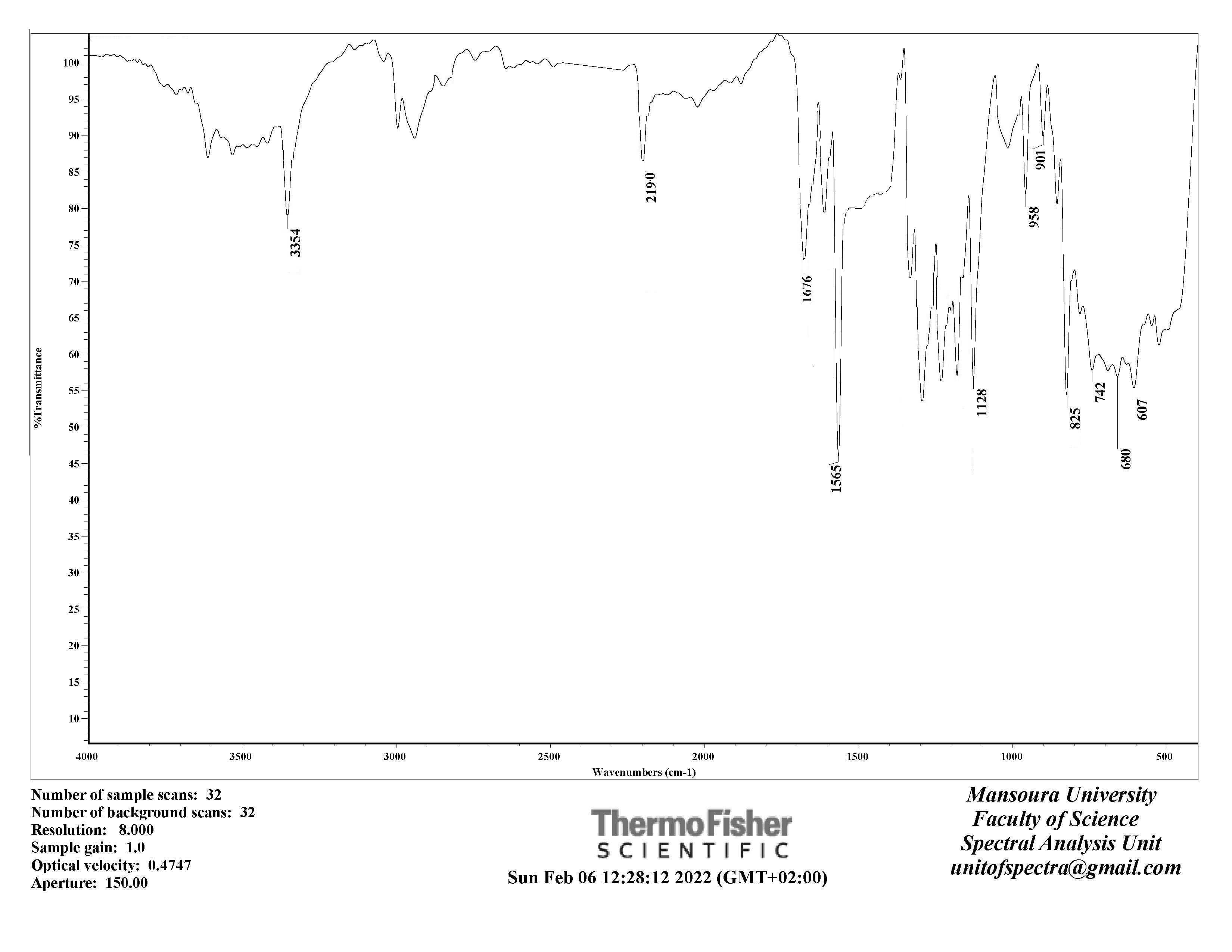


**Figure (S8): IR spectrum of compound AS-2 on TiO_2_.**


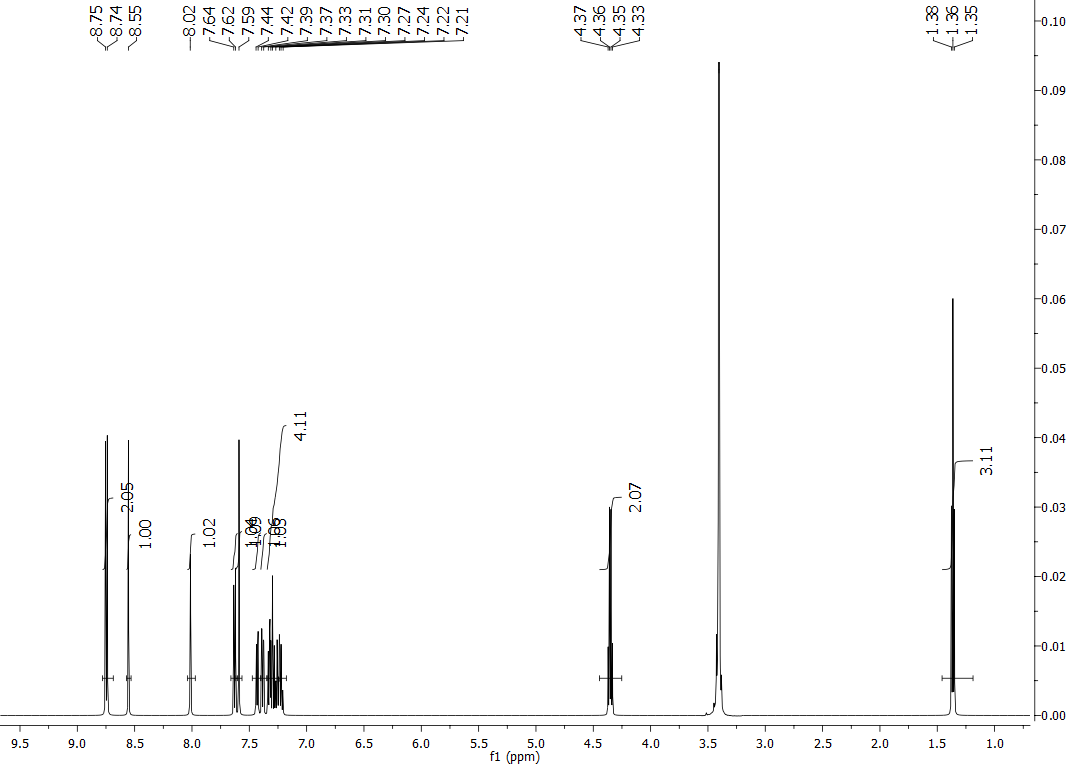


**Figure (S9): ^1^HNMR spectrum of compound AS-2.**


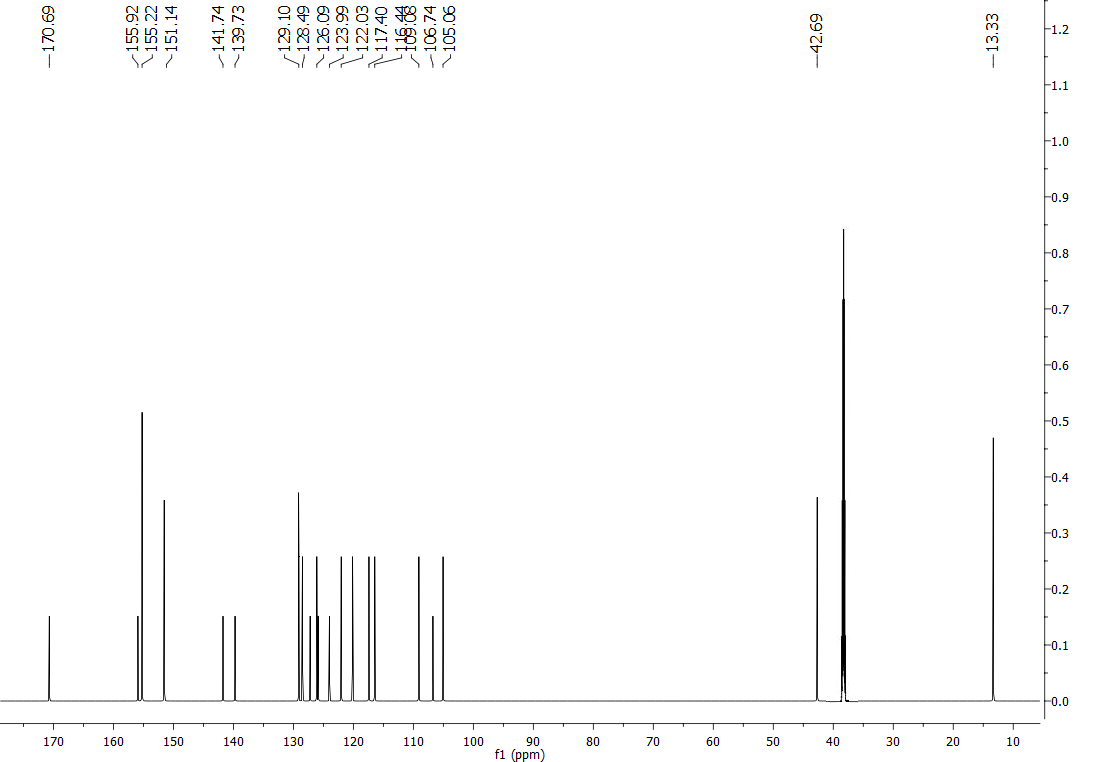


**Figure (S10): ^13^C NMR spectrum of compound AS-2.**

**Figure S11: Mass spectrum of compound AS-2.**


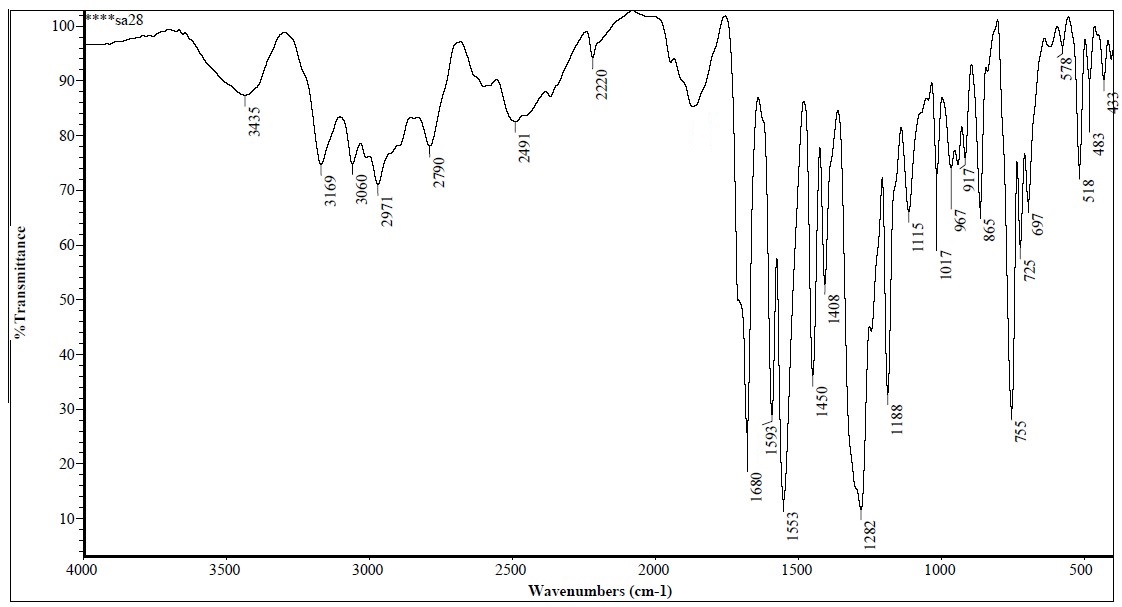


**Figure (S12): IR spectrum of compound AS-3.**


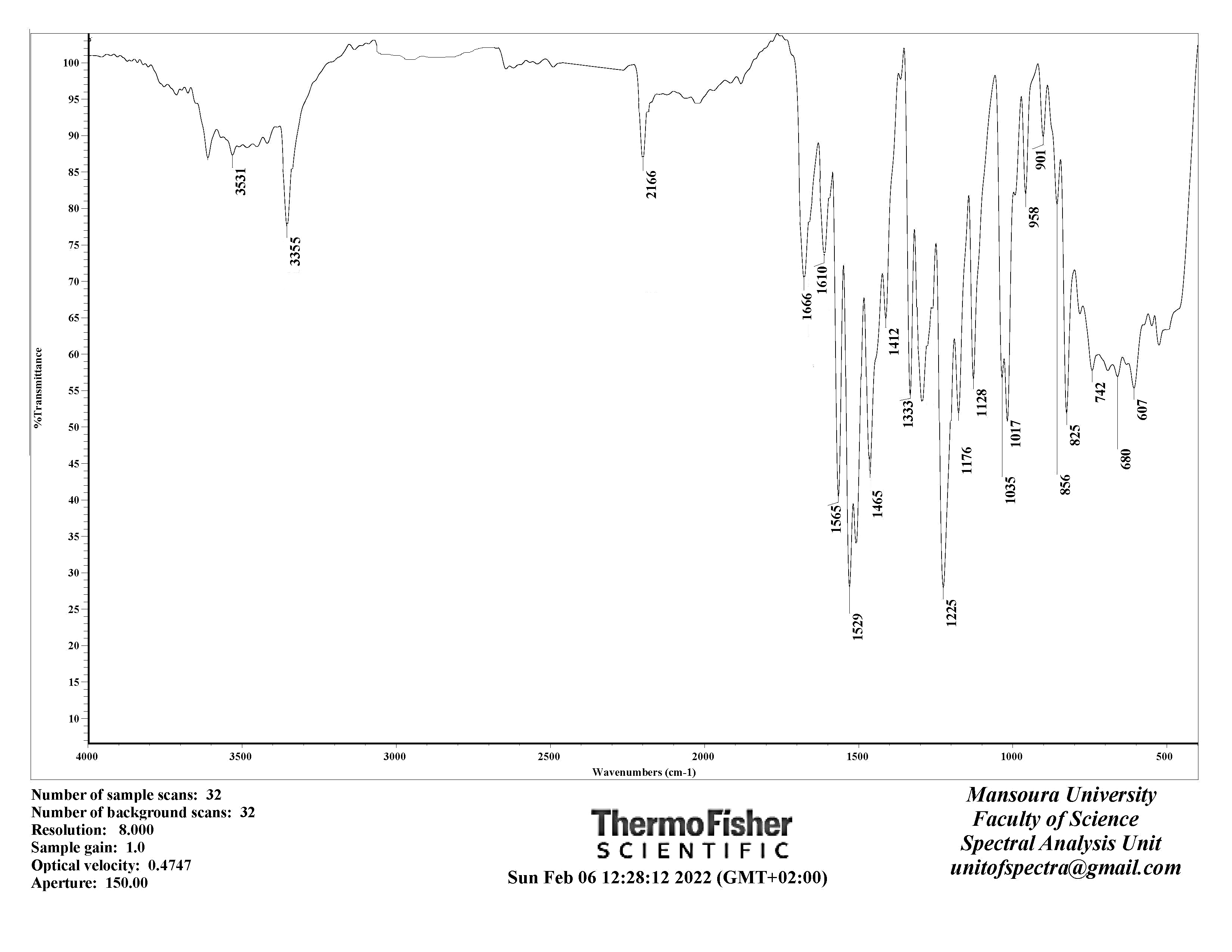


**Figure (S13): IR spectrum of compound AS-3 on TiO_2_.**


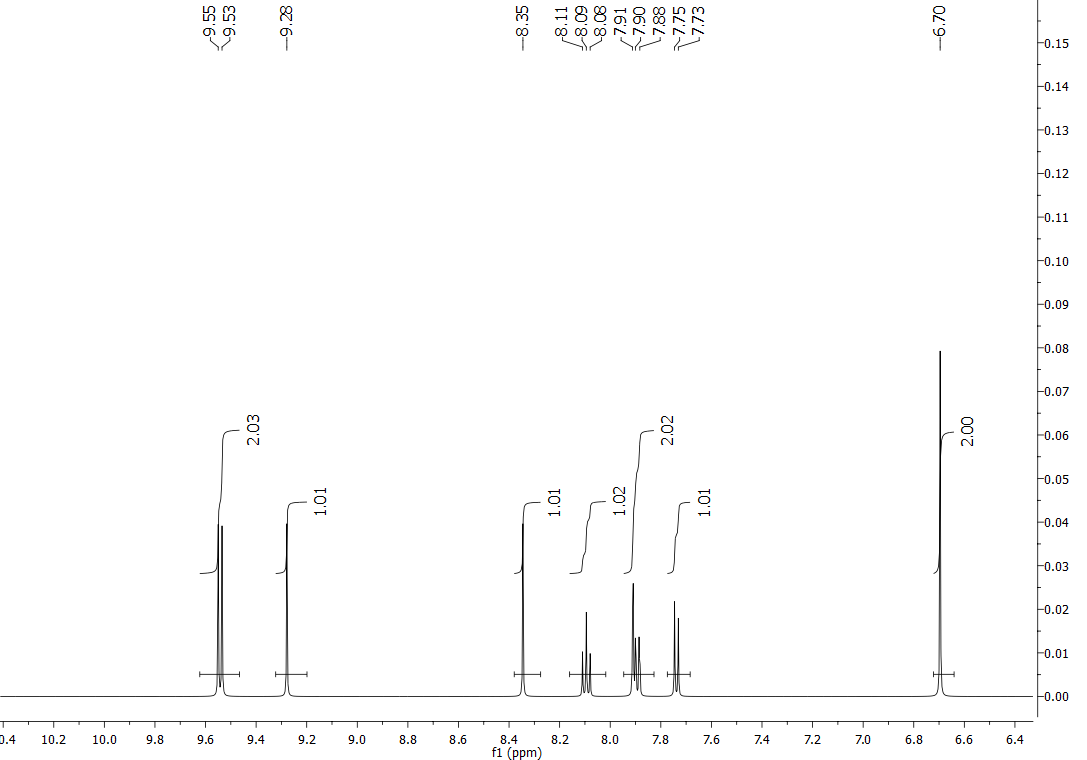


**Figure (S14): ^1^HNMR spectrum of compound AS-3.**


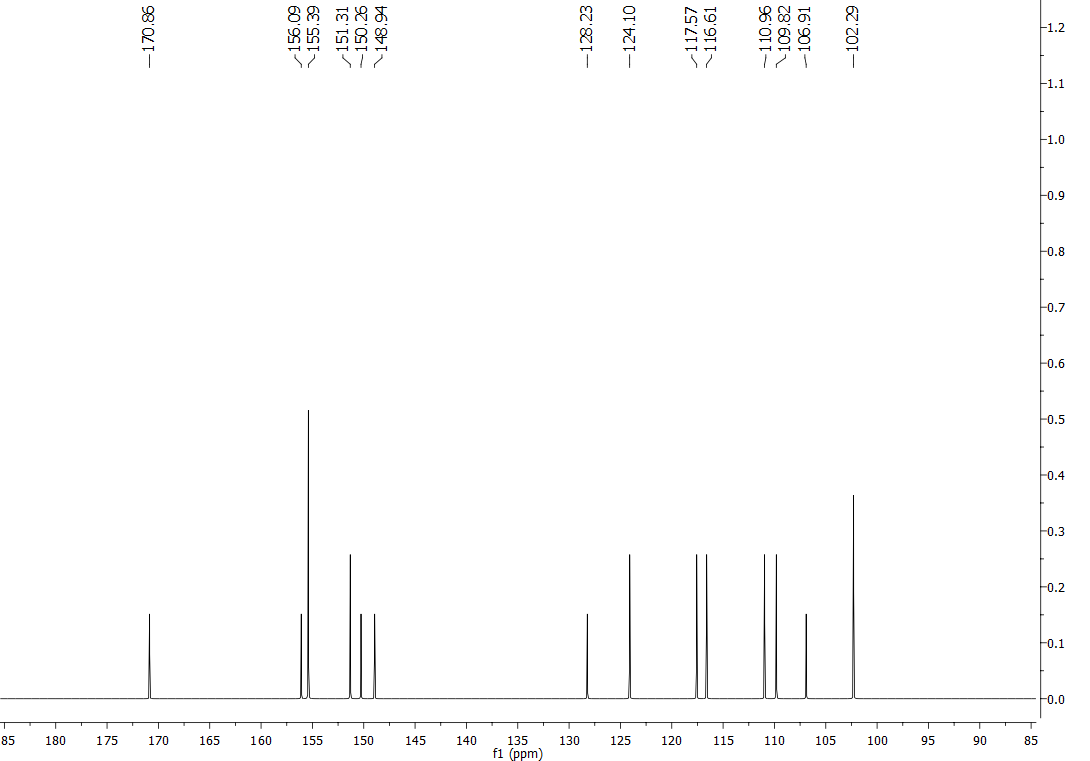


**Figure (S15): ^13^C NMR spectrum of compound AS-3.**

**Figure S16: Mass spectrum of compound AS-3.**


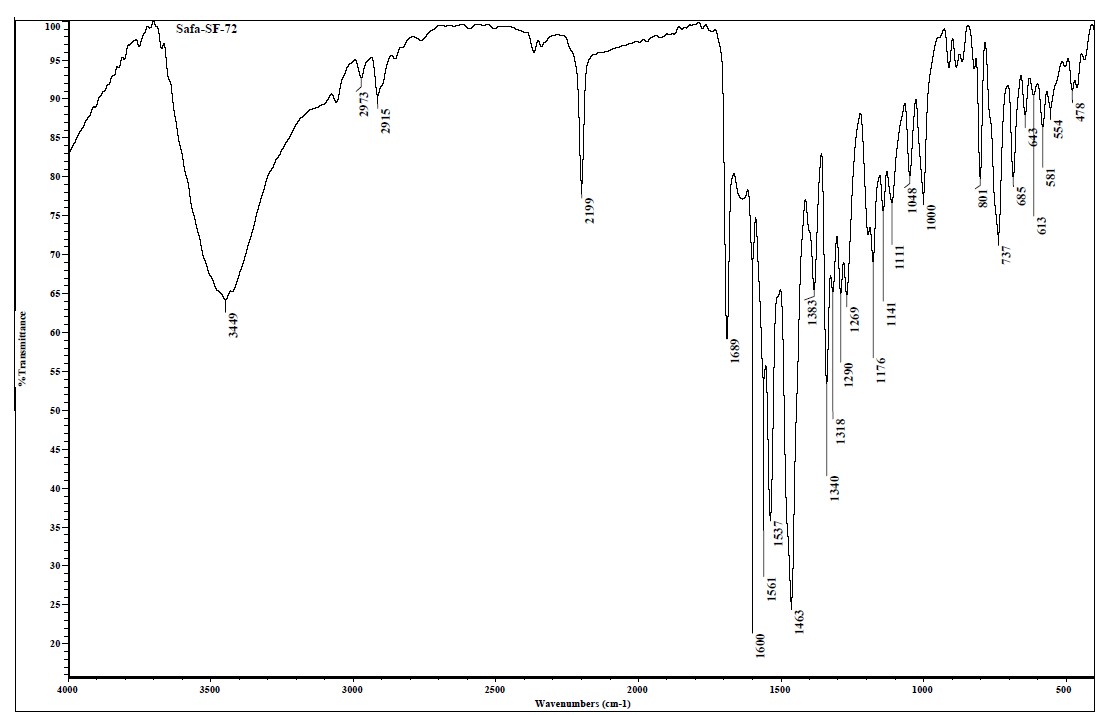


**Figure (S17): IR spectrum of compound AS-4.**

**
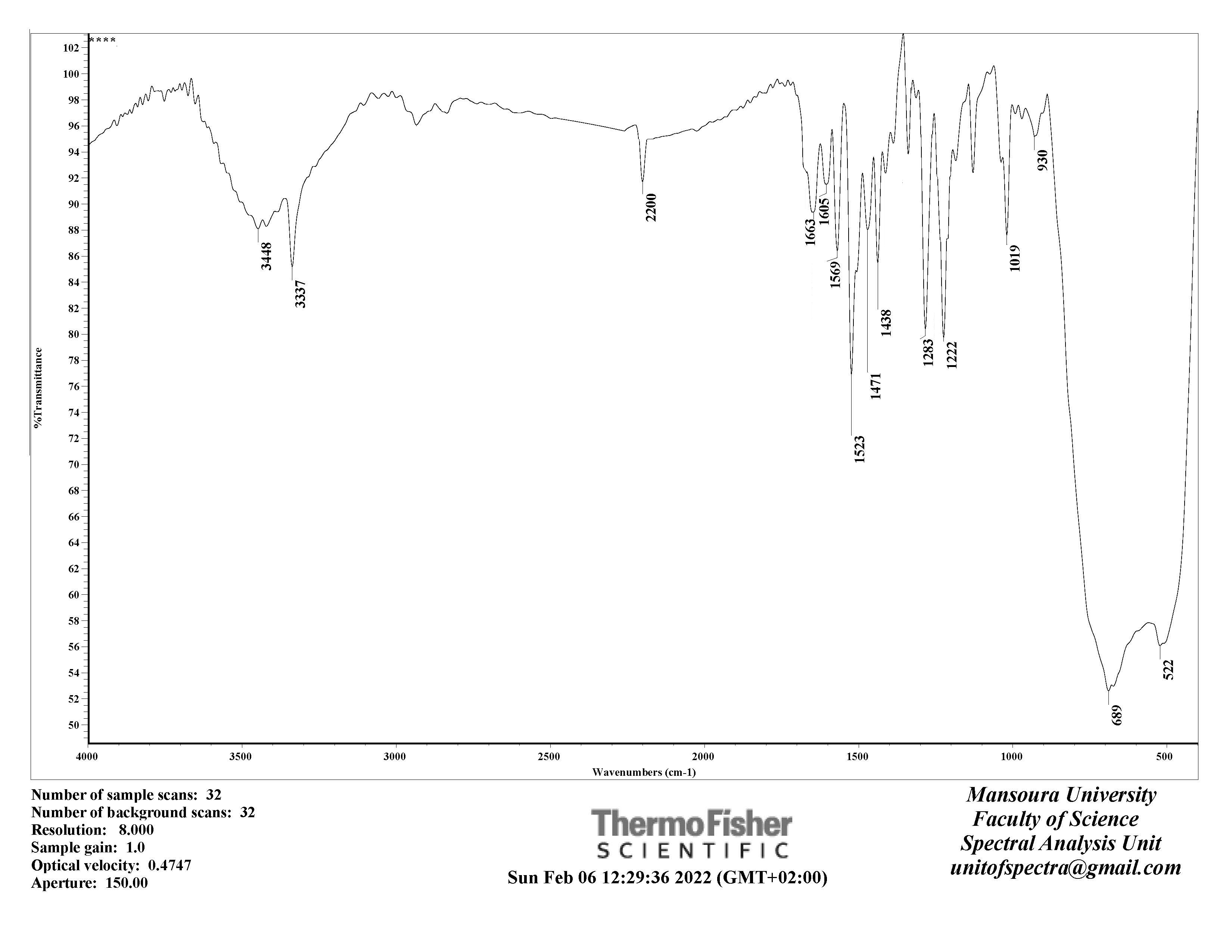
**

**Fig.S18 FTIR spectrum of AS-4 on TiO_2_.**


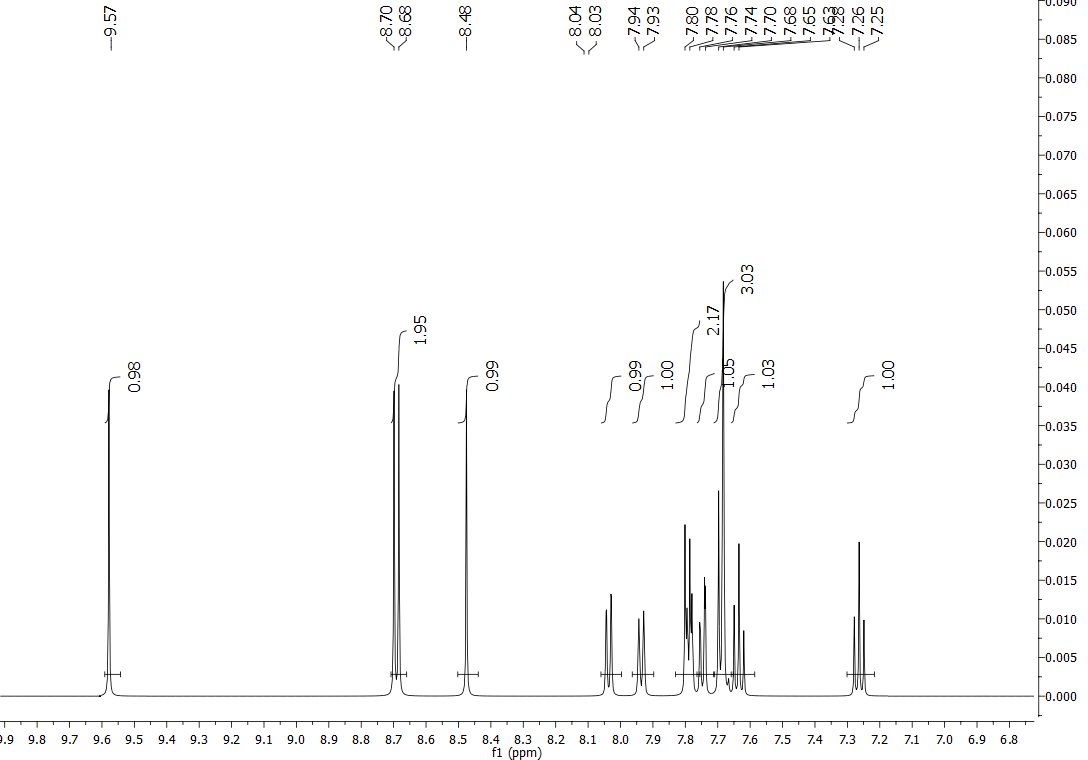


**Figure (S19): ^1^HNMR spectrum of compound AS-4.**


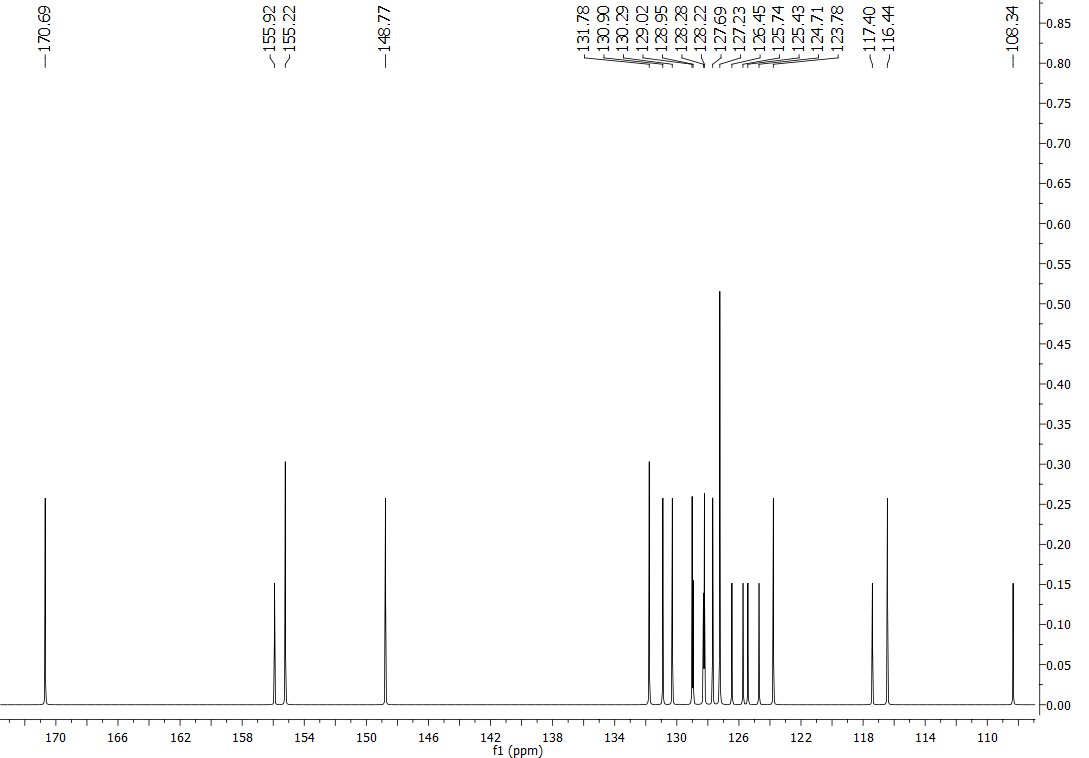


**Figure (S20): ^13^C NMR spectrum of compound AS-4.**

**Figure S21: Mass spectrum of compound AS-4.**

1. **Fabrication process of sensitizers and co-sensitizers.**
2. **Working Electrode (Photoanode-TiO_2_ electrode)**

The fabrication process of dye-sensitized solar cells began with the preparation of a TiO_2_ electrode, which consisted of a double-layer structure with a thickness of 10 + 5 μm. The electrode had a 10 mm thick nano-porous layer and a 5 μm thick scattering layer, which were prepared using a previously reported method [1]. The working electrode is subjected to 15 minutes of sonication in a detergent solution, followed by washing with deionized (DI) water, acetone, and ethanol. The electrode is then treated with TiCl_4_ (60 mM) at 90°C for 60 minutes, and subsequently washed with water and ethanol. A layer of nanoporous TiO_2_ (12-14 μm thick) is printed on the electrode using a single 3M transparent tape. The thickness of the layer is adjusted by punching a hole with a 3/16 5.0 stick. A dispersing layer is printed on top of the TiO_2_ layer using a single 3M transparent tape. The thickness of the layer is adjusted by punching a hole with a 1/4 6.0 stick. The TiO_2_ used for the dispersing layer is R/SP. The printed layers are sintered at 350°C for 10 minutes and then at 500°C for 30 minutes. The electrode is then treated with TiCl_4_ at 90°C for 60 minutes and washed with water and ethanol. The electrode is sintered at 500°C for 30 minutes. In the absence of light, the electrode is submerged in a dye solution for a duration of 20 hours. The organic dye solution is comprised of 0.2 mM of each (**AS-1-4**) in a mixture of acetonitrile, tert-butanol, and DMSO (1:1:1) with addition 0.2 Mm N3. Meanwhile, the ruthenium dye solution consists of 0.2 mM of the dye dissolved in 9 mL solution of consisting of (3 ml) acetonitrile, (3ml) tert-butanol, and (3ml) by ratio (1:1:1). In the case of co-sensitization, the dye solution consists of a mixture of 0.2 mM of the co-sensitizers (**AS-1-4**) and 0.2 mM of the ruthenium dye (N3 dye) in a 9 mL solution consisting of (3 ml) acetonitrile, (3ml) tert-butanol, and (3ml) DMSO by ratio (1:1:1). The performance of the dye-sensitized solar cells was characterized by photovoltaic measurements of sealed cells were made by illuminating the cell through the conducting glass from the anode side with a solar simulator at AM 1.5 illuminations (light intensity: 100 Mw.cm^−2^). The TiO_2_ electrode was treated with a 60 mM TiCl_4_ solution in anhydrous toluene at 90 °C for 60 min. The thickness of each TiO_2_ layer was controlled using a 3/16-inch (≈ 5.0 mm diameter) stainless-steel stick. The TiO_2_ paste designated as R/SP refers to a mixed-phase (rutile/anatase) formulation obtained from Dyesol (Greatcell Solar). The ruthenium sensitizer used for comparison and co-sensitization was N3. The dye solution consisted of 0.2 mM of each AS dye or a mixture of 0.2 mM AS dye and 0.2 mM N3, dissolved in 9 mL of acetonitrile : tert-butanol: DMSO (3: 3:3 v/v/v).
**b) The preparation of a counter electrode involves the following steps:**

The electrode is washed with water, followed by a wash with a 0.1M HCl solution in ethanol (0.2 mL of concentrated HCl in 100 mL of ethanol). The electrode is then subjected to 10 minutes of sonication in an acetone bath. The electrode is dried at 400°C for 15 minutes. A layer of Pt-paste is printed on the electrode using a single 3M transparent tape. The thickness of the layer is adjusted by punching a hole with a 3/8 10.0 stick. The Pt-paste used is Platisol T/SP. The printed layer is cured at 450°C for 10 minutes.

[1] Gad, E. A., Kamar, E. M., & Mousa, M. A. (2020). Experimental and computational study on electronic and photovoltaic properties of chromen-2-one-based organic dyes used for dye-sensitized solar cells. *Egyptian Journal of Petroleum*, *29*(2), 203-209.‏

**Size Range of the Devices and Masking Conditions:**

For all dye-sensitized solar cells (DSCs) tested, including both single-sensitizer and tandem configurations, the active area was consistently measured at approximately 0.25 cm². This uniformity ensures that all devices were evaluated under the same conditions, facilitating a fair comparison of performance metrics such as photocurrent-voltage (J–V) and IPCE. To ensure accurate and reliable *J–V* measurements, a S5 black mask with an oblong aperture of 0.0875 cm² was applied to the devices during the testing process. This mask plays a critical role in defining the working area of the cell, preventing any excess light from reaching the inactive regions or the edges of the devices, which could otherwise introduce variability in the measured current. By restricting the light to a well-defined area of 0.25 cm², we minimized edge effects and ensured that only the active region of the cell contributed to the measured photocurrent. The masking was applied uniformly across all tested solar cells, including the tandem devices. For the tandem DSSCs, which feature two photoactive layers, the mask ensured that both layers were exposed to uniform light intensity, allowing for accurate evaluation of their combined performance.

**2. Calibration and Measurement Conditions of IPCE:**

The incident photon-to-current conversion efficiency (IPCE) measurements were conducted using a QEX10 system (PV Measurements, USA), equipped with a 75 W short arc xenon lamp (UXL-75XE, USHIO, Japan) and a monochromator to selectively illuminate the cells at different wavelengths. Calibration of the incident light was performed before each measurement using a silicon photodiode (IF035, PV Measurements). This step was critical to ensure that the light intensity and spectral output of the source were accurately aligned with the reference photodiode, thereby ensuring reliable IPCE data. For tandem DSSCs, which rely on the complementary absorption profiles of the **N3 Dye** and **AS-1** dyes, the calibration was conducted to ensure that the illumination spectrum covered the relevant wavelength range absorbed by both dyes. The illumination spot size was carefully controlled to be slightly smaller than the active area of the test cells to avoid edge effects and overlap that could skew the measurements. This approach allowed us to assess the individual contributions of each dye in the tandem configuration and accurately capture the overall efficiency of the combined system. In the case of tandem DSSCs, the combination of **AS-1 Dye** (which absorbs primarily in the visible range) and **N3** dyes (which extends absorption into the near-infrared region) required additional considerations in both the calibration and measurement stages. The tandem configuration was optimized to ensure that both the top and bottom layers were illuminated appropriately and that each layer contributed effectively to the overall photocurrent. By masking the device to 0.25 cm² and ensuring uniform light exposure across the two layers, we were able to achieve accurate measurements that reflect the true performance of the tandem devices. The electrochemical impedance spectroscopy measurements were performed to understand carrier transportation behavior and interfacial charge recombination processes in fabricated DSSCs. Currently, EIS analysis is one of the most powerful techniques used to obtain additional information, mainly interfacial reactions of photoexcited electrons in DSSCs. In the present study, EIS spectra were recorded over a frequency range of 100 mHz to 200 kHz at 298K with the Bio-Logic SP-150 impedance analyzer under the illumination under solar illumination using a solar simulator (SOL3A, Oriel) equipped with a 450 W xenon lamp (91160, Oriel). The applied voltage was set at the *V_OC_* of the DSSCs with AC amplitude fixed at 10 mV. The resultant plots were fitted *via* Z-Fit software (Bio-Logic).

Indoor Photovoltaic Measurements:

Indoor photovoltaic measurements were performed under a calibrated warm white, fluorescent light source (Osram, CCT ≈ 3000–3500 K), representative of typical indoor illumination environments. The incident illuminance was set to 1000 lx and measured using a calibrated lux meter, corresponding to an irradiance of approximately 0.283 mW.cm⁻² at the device plane. The spectral power distribution of the lamp is dominated by the visible region (≈400–700 nm), with emission peaks characteristic of phosphor-based fluorescent lighting, which closely matches the absorption window of dye-sensitized solar cells. All indoor J-V measurements were conducted with fixed illumination geometry and stable light intensity to ensure reliable and reproducible device performance evaluation.

**3. Molecular Modeling**

Equilibrium molecular geometries of **AS-1-4** calculated using the Becke's three parameter hybrid functional, Lee–Yang–Parr's gradient corrected correlation functional (B3LYP) and (6-311g(d, p)) [1-3]. The geometry optimization calculations were followed by energy calculations using time-dependent density functional theory (TD-DFT) utilizing the energy, functional B3lyp and the basis set 6-311g (d, p). The solvent (DMF) effect was accounted for by using the conductor-like polarizable continuum model (C-PCM), implemented in Gaussian 09.

**References**

[1] A. D. Becke, Phys. Rev. A **1988**, 38, 3098.

[2]C. T. Lee, W.T. Yang, R.G. Parr, Phys. Rev. B. **1988**, 37, 785

[3] N. Godbout, D.R. Salahub, J. Andzelm, E. Wimmer, Optimization of Gaussian-type basis-sets for local spin-density functional calculations .1. Boron through neon, optimization technique and validation. Can. J. Chem.-Rev. Can. Chim. **1992**, 70, 560-571.

**Tandem fabrication for (AS-1 (Bottom)+N3 Top))**

As depicted in **Fig. S22,** a parallel (PT-DSSC) was constructed utilizing **AS-1,** and **N3 Dye** as sensitizers. This tandem structure incorporates two photoanodes: the upper layer (**FTO/TiO_2_/N3**) was optimized to capture light in the longer wavelength region, whereas the lower layer (**FTO/TiO_2_/AS-1)** effectively absorbed light at shorter wavelengths. The **PT-DSSC** was constructed using two photoanodes connected in parallel: the upper (**FTO/TiO_2/_N3 Dye**) optimized for red-region absorption and the lower (**FTO/TiO_2_/AS-1**) for blue–green absorption. A dual-sided platinum foil served as both counter electrode and interlayer, enabling simultaneous operation of both sub-cells and efficient charge collection through parallel wiring. The electrolyte layer (I^-^/I₃⁻) filled the interspaces between the electrodes, completing the tandem configuration. These two active layers are separated by a dual-sided platinum electrode that facilitates efficient charge transport and suppresses charge recombination. The strategic pairing of N3 Dye with the **N3** mixtures extended the overall light absorption range of the device, thereby improving the photogenerated charge density and enhancing the power conversion efficiency (PCE) of the system ^17^.


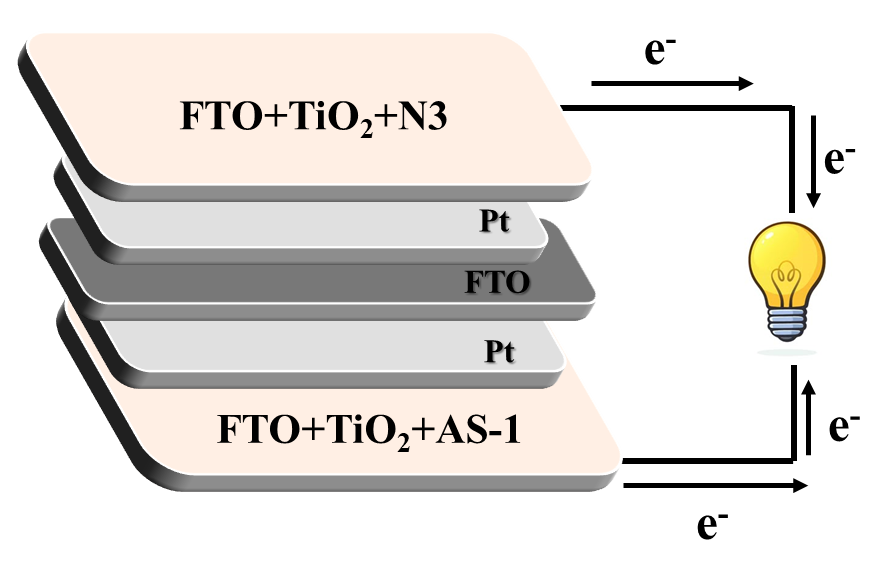


**Fig. S22.** Structure of tandem devices by **AS-1 (Bottom)+** **N3 (Top)** sensitizers

**2.3. *Photovoltaic measurements***

Photovoltaic measurements of sealed cells were made by illuminating the cell through the conducting glass from the anode side with a solar simulator (WXS-155S-10) at AM 1.5 illuminations (light intensity: 100 mW cm^−2^).

**2.4. *Cyclic voltammetry***

Cyclic voltammetry (CV) was performed in DMF with the electrolyte 0.1 M [TBA][PF_6_] at a scan rate of 50 mV s^−1^. The working electrode used is the Glassy carbon, Pt wire represented the counter electrode and the reference electrode is Ag/Ag^+^ in ACN. Fc/Fc^+^ was introduced as internal reference.
